# ZNF768 links oncogenic RAS to cellular senescence

**DOI:** 10.1038/s41467-021-24932-w

**Published:** 2021-08-17

**Authors:** Romain Villot, Audrey Poirier, Inan Bakan, Karine Boulay, Erlinda Fernández, Romain Devillers, Luciano Gama-Braga, Laura Tribouillard, Andréanne Gagné, Éma Duchesne, Danielle Caron, Jean-Sébastien Bérubé, Jean-Christophe Bérubé, Yan Coulombe, Michèle Orain, Yves Gélinas, Stéphane Gobeil, Yohan Bossé, Jean-Yves Masson, Sabine Elowe, Steve Bilodeau, Venkata Manem, Philippe Joubert, Frédérick A. Mallette, Mathieu Laplante

**Affiliations:** 1 Centre de Recherche de l’Institut Universitaire de Cardiologie et de Pneumologie de Québec (CRIUCPQ), Faculté de Médecine, Université Laval, Québec, QC Canada; 2 Centre de Recherche sur le Cancer de l’Université Laval, Université Laval, Québec, QC Canada; 3 Chromatin Structure and Cellular Senescence Research Unit, Maisonneuve-Rosemont Hospital Research Centre, Université de Montréal, Montréal, QC Canada; 4 Département de Biochimie et Médecine Moléculaire, Université de Montréal, Montréal, QC Canada; 5 Centre de Recherche du Centre Hospitalier Universitaire de Québec, Université Laval, Québec, QC Canada; 6 Centre de Recherche du CHU de Québec—Université Laval, Axe Oncologie, Québec, QC Canada; 7 Centre de Recherche en Données Massives de l’Université Laval, Québec, QC Canada; 8 Département de Biologie Moléculaire, Biochimie Médicale et Pathologie, Faculté de Médecine, Université Laval, Québec, QC Canada; 9 Département de Médecine, Université de Montréal, Montréal, QC Canada

**Keywords:** Cancer, Growth factor signalling, Senescence

## Abstract

RAS proteins are GTPases that lie upstream of a signaling network impacting cell fate determination. How cells integrate RAS activity to balance proliferation and cellular senescence is still incompletely characterized. Here, we identify ZNF768 as a phosphoprotein destabilized upon RAS activation. We report that ZNF768 depletion impairs proliferation and induces senescence by modulating the expression of key cell cycle effectors and established p53 targets. ZNF768 levels decrease in response to replicative-, stress- and oncogene-induced senescence. Interestingly, ZNF768 overexpression contributes to bypass RAS-induced senescence by repressing the p53 pathway. Furthermore, we show that ZNF768 interacts with and represses p53 phosphorylation and activity. Cancer genomics and immunohistochemical analyses reveal that ZNF768 is often amplified and/or overexpressed in tumors, suggesting that cells could use ZNF768 to bypass senescence, sustain proliferation and promote malignant transformation. Thus, we identify ZNF768 as a protein linking oncogenic signaling to the control of cell fate decision and proliferation.

## Abstract (web summary)

RAS-induced senescence is a safeguarding process against tumour development. Here, the authors show that RAS activation destabilises the transcription factor ZNF768, which blocks ZNF768- dependent repression of p53 activity and thus induces senescence.

## KEY FINDINGS

- ZNF768 is phosphorylated and degraded upon RAS activation
- ZNF768 depletion impairs proliferation and causes cellular senescence
- ZNF768 binds and represses p53 and its overexpression suffices to bypass senescence
- Elevated ZNF768 levels in human tumors may serve to avoid cellular senescence and support proliferation

## Introduction

RAS proteins (HRAS, NRAS, and KRAS) are small GTPases that lie upstream of a broad signaling network controlling proliferation. These proteins are often mutated and hyperactive in tumor cells^1^. Nearly 30% of human cancers harbor mutations in *RAS* genes. In response to mitogens, RAS activates the phosphoinositide-3-kinase (PI3K) and the mitogen-activated protein (MAPK) pathways^1^. When active, these two signaling nodes promote cell growth by stimulating various processes including protein, lipid and nucleotide biosynthesis^2,3^. The rise in cell mass linked to the activation of these anabolic processes is a critical feature allowing cell cycle entry and normal cell division. PI3K and MAPK activation also drive proliferation by promoting cell cycle progression and by repressing apoptosis through the phosphorylation of numerous effectors^4–6^. Supporting the role of RAS signaling in promoting cell growth and proliferation, constitutive activation of this pathway was shown to promote the development of various types of cancer in mice^7^.

Although RAS signaling is important to support proliferation, unrestrained RAS activation in primary mammalian cells typically triggers a cascade of molecular and cellular events leading to cellular senescence, a state of permanent cell cycle arrest in which cells remain metabolically active^8^. This process, termed oncogene-induced senescence, has emerged as an important cancer-protective response to oncogenic events, serving to eliminate early neoplastic cells^9–12^. Hyperactive RAS promotes cellular senescence through several complementary routes, which can differ depending on the cellular context. It is generally accepted that oncogenic RAS triggers cellular senescence by activating p53 and p16^INK4^-Rb pathways, by promoting the degradation of pro-proliferation proteins and by activating the DNA damage response^8,13–16^. Bypass or evasion from cell senescence has been proposed as a pivotal step in the pre-neoplastic phase leading to cancer^17,18^.

How exactly cells integrate RAS signaling to balance cellular senescence and proliferation is not well understood. Furthermore, the precise mechanisms by which cells support hyperactive RAS signaling to bypass cellular senescence are incompletely characterized. Here, we identify the transcription factor ZNF768 as a protein phosphorylated and destabilized upon RAS activation. We show that ZNF768 depletion impairs proliferation and rapidly induces cellular senescence by modulating the expression of key cell cycle effectors and p53 target genes. We found that ZNF768 levels are reduced in response to replicative-, stress- and oncogene-induced senescence and that ectopic expression of ZNF768 contributes to bypass of RAS-induced senescence, by hindering p53 activation. We then show that ZNF768 physically interacts with p53 to repress its phosphorylation and its activity. Cancer genomics and immunohistochemical analyses revealed that ZNF768 is frequently amplified and/or overexpressed in various human malignancies, suggesting that ZNF768 could contribute to the bypass of cellular senescence and to the promotion of oncogene-induced transformation. Thus, we identify ZNF768 as a target of RAS linking growth factor signaling to the control of cell proliferation.

## Results

### ZNF768 is a phosphoprotein destabilized upon RAS activation

Analyses of phosphoproteomics studies indicate that ZNF768 is part of a small group of uncharacterized transcriptional regulators potentially phosphorylated downstream of RAS (Supplementary Fig. 1A and Supplementary Data 1)^19^. ZNF768 is a 540-amino acid protein conserved in mammals (Supplementary Fig. 1B). This protein contains C2H2 domains in its C-terminal section and localizes to the nucleus (Supplementary Fig. 1B, C). A unique feature of ZNF768 is the presence of amino acid stretches at the N-terminal end resembling the heptapeptide repeats found in the C-terminal domain (CTD) of the large subunit (RPB1) of RNA polymerase II (Pol II) (Supplementary Fig. 1D). Strikingly, large-scale protein sequence analyses using InterPro revealed that, beyond RPB1, ZNF768 is the only human protein containing such repeats (Supplementary Fig. 1E). In RPB1, these repeats are dynamically phosphorylated by various kinases, which play fundamental roles in regulating RPB1 function^20,21^.

**Fig. 1:**
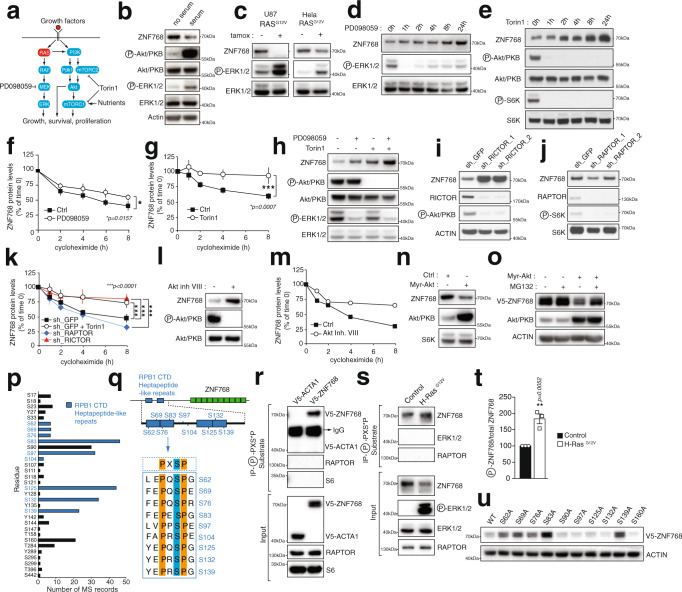
Identification of ZNF768 as a phosphoprotein destabilized upon RAS activation. **A** Overview of the RAS signaling pathway. **B** RPE cells were incubated with or without 10% serum for 2 h and western blots were performed. **C** Cells were transduced to overexpress RAS^G12V^ with a tamoxifen inducible system. Cells were exposed to tamoxifen (100 µM) for 3 days and western blots were performed. **D**, **E** U87 cells were treated with **D** PD098059 (50 µM) or **E** Torin1 (250 nM) and western blots were performed. **F**, **G** U87 cells were pretreated with **F** PD098059 (50 µM, 4 h) or **G** Torin1 (250 nM, 12 h) and then treated with cycloheximide (1 µg/ml). The result presented is the average of *n* = 3 (PD098059) and *n* = 4 (Torin1) independent experiments. **H** U87 cells were treated with PD098059 (50 µM), Torin1 (250 nM), or a combination of both drugs for 3 and 1 h, respectively and western blots were performed. **I**, **J** U87 cells were transduced to knockdown **I** RICTOR or **J** RAPTOR and western blots were performed. **K** Cells prepared as described in (**I**, **J**) were treated with cycloheximide as described in (**F**). The results were produced from at least six independent biological replicates per conditions (*n* = 6). **L** Hela cells were treated with Akt inhibitor VIII (50 µM) for 24 h and western blots were performed. **M** U87 cells were treated with Akt inhibitor VIII (50 µM) for 12 h and then treated with cycloheximide as described in (**F**). The results presented is the average of two experiments (*n* = 2). **N** Hela cells were transduced to overexpress a control protein or myr-Akt and western blots were performed. **O** Cells described in (**N**) were transduced to express V5-ZNF768 and treated or not with MG132 (20 µM) for 3 h and western blots were performed. **P** Phosphosite analyses showing the number of MS records for each serine residue found in ZNF768. **Q** Structure of ZNF768 protein and phosphorylation motifs found in the CTD domain. **R** U87 cells were transduced to overexpress V5-ACTA1 or V5-ZNF768. Immunoprecipitations using an antibody raised against PXS*P were performed and protein analyzed by western blot. **S**, **T** V5-ZNF768 and H-RAS^G12V^ were overexpressed in Hela cells. Immunoprecipitations and western blots were performed as described in (**R**). The results presented is the average of three experiments (*n* = 3). **U** U87 cells were transduced to overexpress ZNF768 phosphomutants and western blots were performed. In all panels, data represent the mean ± SEM. In panel **F**, **G** and **K**, significance was determined by two-way ANOVA. In panel **T**, significance was determined by two-tailed, unpaired *t* test. Details about reproducibility are provided in the Statistics and reproducibility included in the Methods section. Source data are provided as a Source data file.

To define the relation between RAS activation and ZNF768, we first tested the impact of serum treatment on ZNF768 protein levels in normal, RPE cells (Fig. 1A, B). Serum stimulation was associated with activation of both MAPK and PI3K signaling and caused a significant decrease in ZNF768 levels (Fig. 1B). ZNF768 was also destabilized in cells expressing a constitutively active form of RAS (RAS^G12V^) (Fig. 1C). Supporting the connection between RAS activation and ZNF768, we found that inhibition of downstream effectors of RAS signaling such as MAP/ERK kinase 1 (MEK1) or the mechanistic target of rapamycin (mTOR) with PD098059 (Fig. 1D and Supplementary 1F) or Torin1 (Fig. 1E and Supplementary Fig. 1G), respectively, led to a rise in ZNF768. Importantly, these effects were not associated with changes in *ZNF768* mRNA expression (Supplementary Fig. 1H) but were rather linked to increased ZNF768 protein stability (Fig. 1F, G). We also observed that the effects of MEK and mTOR inhibitors on ZNF768 were additive, indicating that both MAPK and PI3K signaling control ZNF768 stability through parallel and complementary mechanisms (Fig. 1H).

The mTOR kinase nucleates two complexes known as mTORC1 and mTORC2 (Fig. 1A). To better define which mTOR complex affects ZNF768 stability downstream of RAS/PI3K, RAPTOR, or RICTOR were depleted in order to inhibit the action of mTORC1 and mTORC2, respectively. These experiments revealed that inhibition of mTORC2 (Fig. 1I), but not mTORC1 (Fig. 1J), promotes ZNF768 stability (Fig. 1K). Supporting these findings, inhibition of mTORC1 with rapamycin had no effect on ZNF768 protein levels in all tested cell lines (Supplementary Fig. 1I). Protein kinase B/Akt (Akt) is directly phosphorylated on serine 473 by mTORC2 and serves as one of its major downstream effectors^22^. We found that both pharmacological and genetic inactivation of Akt were sufficient to stabilize ZNF768 (Fig. 1L, M and Supplementary Fig. 1J). Conversely, constitutive activation of Akt reduced ZNF768 protein levels in various cell types (Fig. 1N and Supplementary Fig. 1K). In follow-up experiments, we found that proteasome inhibition with MG132 was sufficient to prevent Akt-mediated degradation of ZNF768 (Fig. 1O). Collectively these experiments indicate that hyperactive growth factor signaling destabilizes ZNF768 by promoting its degradation by the proteasome.

We next turned to the online tool PhosphoSitePlus to identify the residues in ZNF768 that were previously reported to be phosphorylated in large-scale phosphoproteomics studies. As shown in Fig. 1P, 32 sites were identified in at least one mass spectrometry (MS) reference. Strikingly, we found that many of these residues fell within, or near to the heptad repeats stretches in the N-terminal end of ZNF768 (Fig. 1Q). To confirm that ZNF768 is phosphorylated on these residues, we performed IPs using anti-PXS*P antibody and probed for ZNF768 by western blot. Using this approach, we showed that ZNF768 is phosphorylated on these motifs (Fig. 1R), and that oncogenic RAS^G12V^ increases ZNF768 phosphorylation (Fig. 1S, T). Supporting the importance of phosphorylation for the regulation of ZNF768 stability, we found that serine to alanine mutations on residues S62, S69, S76, S83, and S139 increased ZNF768 stability (Fig. 1U). Altogether, these results identify ZNF768 as a phosphoprotein whose stability is reduced upon RAS activation.

### ZNF768 depletion blocks proliferation and induces senescence-like features

**Fig. 2:**
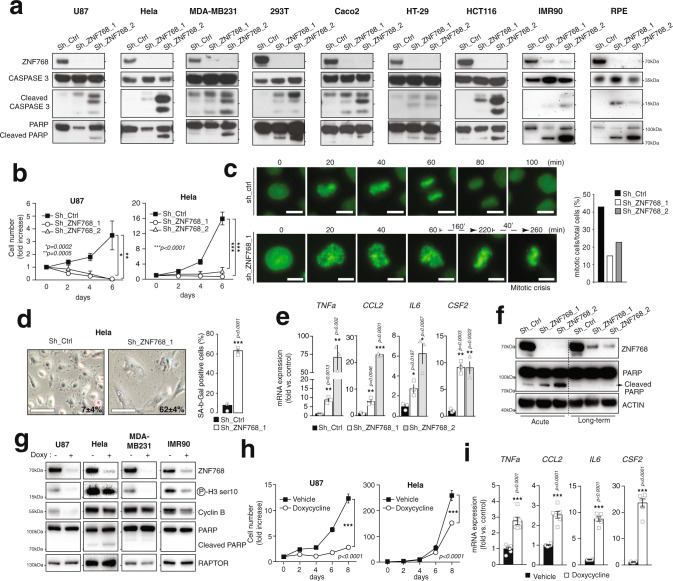
ZNF768 depletion blocks proliferation and induces senescence-like features. **A** Cells were transduced with lentiviruses expressing shRNA to knockdown ZNF768. Cells were selected and western blots were performed. **B** U87 and Hela cells were treated as described in (**A**) and counted. The result presented is the average of three independent experiments (*n* = 3). **C** Hela cells were transduced with lentiviruses expressing shRNA to knockdown ZNF768. The cells were stained with SiR-DNA and used in live-imaging microscopy 48 h post infection. The mitotic index was calculated from one experiment (Scale: 5 µm). **D** Hela cells were treated as described in (**A**) and SA-β-gal staining was performed. The % of SA-β-gal positive cells is presented (*n* = 3/condition) (Scale: 100 µm). **E** U87 cells were transduced and selected as described in (**A**). RNA was harvested 96 h post-infection and the expression of SASP genes was measured by RT-qPCR (*n* = 3/condition). **F** HCT116 cells were transduced to knockdown ZNF768, as described in panel (**A**). Protein lysates were harvested 2 days (Acute) or 2 weeks (long-term) post-infection and western blots were performed. **G** Cells were transduced with lentiviruses allowing the conditional expression of a shRNA targeting ZNF768. After selection with puromycin, cells were treated or not with doxycycline (20 ng/µl) for 48 h to induce the acute knockdown of ZNF768 and western blots were performed. **H** Inducible U87 and Hela cells described in (**G**) were plated, treated or not with doxycycline (20 ng/µl) and counted (U87; *n* = 2/condition. Hela; *n* = 3/condition). **I** Inducible U87 cells were treated or not for 7 days with doxycycline (20 ng/µl). RNA was harvested and the expression of SASP genes was measured by RT-qPCR (*n* = 6/condition). In all panels, data represent the mean ± SEM. In panel **B** and **H**, significance was determined by two-way ANOVA. In panel **D**, **E**, and **I**, significance was determined by two-tailed, unpaired *t* test. Details about reproducibility are provided in the Statistics and reproducibility included in the Methods section. Source data are provided as a Source data file.

In a first attempt to characterize ZNF768 functions, normal and cancer cell lines of different origins were transduced with lentiviruses expressing short-hairpin RNA (shRNA) to deplete ZNF768. Testing the impact of ZNF768 depletion in many cell lines was achieved to insure the phenotypes linked to ZNF768 depletion were not cell line-specific. Although ZNF768 knockdown was initially well tolerated following lentiviral infection, a rise in cell death progressively took place few days post-selection. This effect was associated with an induction of cleaved Caspase 3 and cleaved poly(ADP-ribose) polymerase-1 (PARP) levels (Fig. 2A), a rise in the expression of proapoptotic genes (Supplementary Fig. 2A), and a reduction in cell number (Fig. 2B). Live-cell imaging revealed that ZNF768 loss reduced the mitotic index and increased mitotic catastrophes, further confirming the proliferation defects associated with ZNF768 depletion (Fig. 2C and Supplementary Fig. 2B). While performing the above experiments, we observed that some cells did not die following ZNF768 depletion. A portion of the cells surviving the acute loss of ZNF768 showed clear signs of cellular senescence, including enlarged flattened shape (Supplementary Fig. 2C to 2E), increased senescence-associated β-galactosidase activity (SA-β-gal) (Fig. 2D and Supplementary Fig. 2F), and high expression of genes that are part of the senescence-associated secretory phenotype (SASP) (Fig. 2E). Interestingly, we also observed that a small fraction of cells surviving ZNF768 depletion could still proliferate and be amplified when cultures were maintained over a longer period of time. As shown in Fig. 2F, these cells displayed higher ZNF768 levels compared to the cells collected at early time points following lentiviral infection, indicating that a minimal level of ZNF768 is required to sustain proliferation in vitro.

In order to better define the phenotypes linked to ZNF768 depletion, we next generated doxycycline-inducible lentiviral vectors to acutely control the timing and the intensity of ZNF768 knockdown. As shown in Supplementary Fig. 2G, this inducible approach led to a less severe depletion of ZNF768. Partial ZNF768 depletion did not induce apoptosis in any of the cell lines tested (Fig. 2G and Supplementary Fig. 2H). However, we found that lowering ZNF768 levels was sufficient to reduce Cyclin B expression, histone H3 phosphorylation and cell number (Fig. 2G, H and Supplementary Fig. 2H, I). Cells partially depleted from ZNF768 were enlarged, displayed high levels of SA-β-gal activity and showed a significant rise in the expression of established SASP markers (Fig. 2I and Supplementary Fig. 2J, K). No signs of apoptosis were measured in these cells (Supplementary Fig. 2L). Importantly, these phenotypes were maintained several days after removing doxycycline, indicating that ZNF768 depleted cells remained arrested even after stopping ZNF768 knockdown (Supplementary Figs. 2M–P). Altogether, these results show that reducing ZNF768 levels is sufficient to trigger senescence in normal and cancer cell lines in vitro.

### ZNF768 depletion affects the expression of key genes controlling proliferation and senescence

**Fig. 3:**
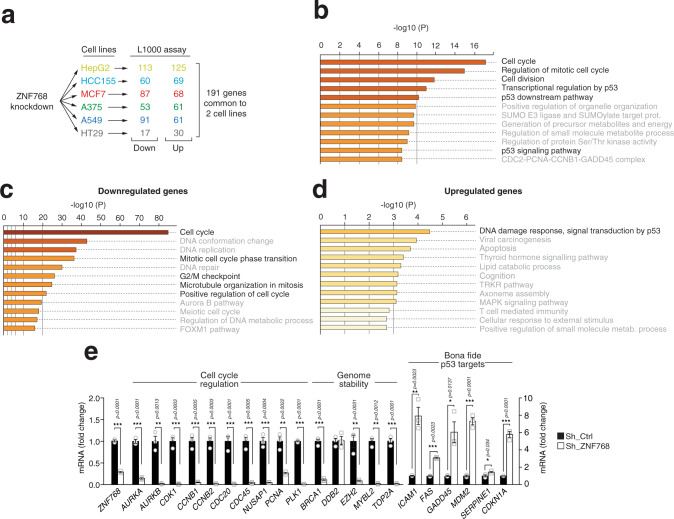
ZNF768 depletion affects the expression of key genes controlling proliferation and senescence. **A** Schematic overview of the approach designed to test the effect of ZNF768 depletion in various cell lines (HT29, A375, A549, MCF7, HCC155, and Hepg2) using the iLincs resource. **B** Gene ontology analysis performed with Metascape of the list of genes regulated in at least two cell lines in the analysis described in (**A**). The full list of gene is presented in Supplementary Data 2. **C**, **D** U87 cells were transduced with lentiviruses to knockdown ZNF768. Cells were selected and RNA was isolated 96 h post infection. A microarray was performed from the isolated RNA (*n* = 3/condition). Gene ontology analysis performed with Metascape on the list of genes identified by microarray that were (**C**) downregulated or (**D**) upregulated in response to ZNF768 depletion. The full list of genes is presented in Supplementary Data 4 and 5. **E** RT-qPCR analysis of gene expression in the samples prepared in (**C**, **D**) (*n* = 3/condition). In all panels, data represent the mean ± SEM. In panel **E**, significance was determined by two-tailed, unpaired *t* test. Details about reproducibility are provided in the Statistics and reproducibility included in the Methods section. Source data are provided as a Source data file.

ZNF768 is a transcription factor that binds mammalian-wide interspersed repeats (MIRs) to control the expression of genes in a cell-specific manner^23^. To understand how ZNF768 modulates proliferation and cellular senescence, we first looked at the gene expression profile upon ZNF768 depletion in 6 different cell lines using iLincs, a publicly available resource providing the expression profile of almost 1000 genes (L1000 assay) in response to various perturbagens^24^. Although ZNF768 depletion affected gene expression differentially in every cell line, 191 genes were found to be similarly regulated in at least 2 of them (Fig. 3A and Supplementary Data 2). Comprehensive analysis of this gene signature with Metascape revealed a significant enrichment for genes linked to the cell cycle and to the p53 signaling pathway (Fig. 3B and Supplementary Data 2). To extend the analysis of the gene signature linked to ZNF768 depletion, a genome-wide transcriptomics analysis was performed in U87 cells following ZNF768 knockdown. U87 cells were selected because this cell line was shown to be highly sensitive to ZNF768 depletion (Fig. 2B). These experiments were performed rapidly following ZNF768 knockdown in order to characterize the primary effects linked to its cellular depletion (Supplementary Fig. 3A and Supplementary Data 3). Confirming the above findings, we found that the genes affected upon ZNF768 knockdown were also functionally linked to the cell cycle and p53 signaling (Fig. 3C, D and Supplementary Data 4 and 5). For instance, genes coding for proteins playing roles in cell cycle regulation (ex. *CDK1*, *MYBL2*, *CCNB1*, *CCNB2*), chromosome segregation (e.g., *NUSAP1*, *AURKA*, *AURKB*, *PLK1*, and *CDC20*), genome replication (*PCNA*, *CDC45*, and *TOP2A*) and genome stability (e.g., *BRCA1*, *DDB2*, *EZH2*, *MYBL2*, and *TOP2A*) were severely repressed in ZNF768 depleted cells (Fig. 3E and Supplementary Data 4). Also, many *bona fide* p53-target genes were significantly upregulated in these cells (e.g., *ICAM1*, *FAS*, *GADD45A*, *MDM2*, *SERPINE1*, and *CDKN1A/p21*) (Fig. 3E and Supplementary Data 5). Importantly, the effects of ZNF768 depletion on the expression of most of these genes were reproduced in cells acutely and partially depleted from ZNF768 using doxycycline-inducible cell lines (Supplementary Fig. 3B). In these experiments, the reduction in the expression of genes regulating the cell cycle preceded the transcriptional changes in p53 targets, indicating that ZNF768 depletion sequentially affects gene expression and that the transcription of cell cycle-related genes likely occurs independently of p53. Supporting this hypothesis, we found that ZNF768 depletion repressed the expression of cell cycle-related genes and impaired proliferation to the same extent in p53^+/+^ and p53^−/−^ cells (Supplementary Fig. 3C–E). Interestingly, we measured more SA-β-gal positive cells and higher *CDKN1A/p21* expression in p53^+/+^ cells following ZNF768 knockdown (Supplementary Fig. 3F–H), arguing that the induction of cellular senescence in ZNF768 depleted cells strongly depends on p53. Altogether, these results indicate that ZNF768 controls proliferation through p53-dependent and independent processes.

### ZNF768 is degraded upon senescence entry

**Fig. 4:**
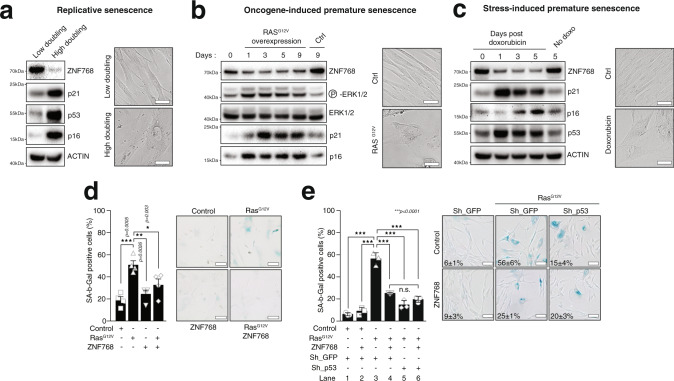
ZNF768 is depleted upon senescence entry and its overexpression contributes to bypass this process. **A** Protein lysates were prepared from IMR90 cells at Low or High population doublings. Western blot analyses were performed for the indicated proteins. Representative pictures of the cells are shown on the right part of the panel (Scale: 50 µm). **B** IMR90 cells were transduced with lentiviruses to overexpress a constitutively active form of RAS^G12V^. Protein lysates were prepared at the indicated time following the overexpression and western blot analyses performed for the indicated proteins. Representative pictures of the cells are shown on the right part of the panel (Scale: 50 µm). **C** IMR90 cells were treated overnight with doxorubicin (0.1 µM). The cells were next washed and followed for the indicated times. Western blot analyses were performed for the indicated proteins. Representative pictures of the cells are shown on the right part of the panel (Scale: 50 µm). **D** Primary MEFs were transduced with retroviruses expressing different combinations of FLAG-ZNF768, RAS^G12V^, or empty vector. After antibiotic selection, cells were maintained in culture until the first signs of senescence bypass were visible. Cells were then fixed and SA-β-gal assays were performed. The percentage of SA-β-gal positive cells is shown. The result presented is the average of four independent experiments (*n* = 4). Representative images are shown (Scale: 100 µm). **E** IMR90 cells were transduced with retroviruses expressing different combinations of FLAG-ZNF768, shGFP, shp53, RAS^G12V^, or empty vector. After antibiotic selection, cells were maintained in culture for 8 days. Cells were then fixed and SA-β-gal assays were performed. The percentage of SA-β-gal positive cells from three independent counts are indicated and representative images are shown for each condition (Scale: 100 µm). In panels **D** and **E**, mean values were compared through a one-way ANOVA with Tukey’s multiple-comparisons test. Details about reproducibility are provided in the Statistics and reproducibility included in the Methods section. Source data are provided as a Source data file.

To define the relationship between ZNF768, cellular senescence and proliferation, we measured ZNF768 protein levels in response to replicative and premature senescence (including oncogene- and stress-induced senescence). To first test the regulation of ZNF768 in response to replicative senescence, protein lysates were prepared from normal human diploid fibroblasts IMR90 cultivated after either low or high population doublings. IMR90 are routinely used to study replicative senescence^25^. As expected, we observed increased levels of senescence-associated markers in IMR90 cells cultivated for numerous passages (Fig. 4A). A significant decrease in ZNF768 protein levels was observed during replicative senescence (Fig. 4A). To test whether ZNF768 was similarly repressed in response to premature senescence, we overexpressed oncogenic RAS^G12V^ in IMR90 cells using a tamoxifen-inducible system and followed these cells over 9 days. Cells overexpressing RAS^G12V^ were morphologically elongated and flat, failed to reach confluence and expressed high levels of p21 and p16 (Fig. 4B). Interestingly, ZNF768 levels were rapidly reduced upon RAS expression, further supporting a connection between ZNF768 depletion and cellular senescence entry (Fig. 4B). We next tested the regulation of ZNF768 during stress-induced senescence triggered by the DNA damaging agent doxorubicin. IMR90 and HCT116 cells were treated overnight with a low dose of doxorubicin (0.1 µM) before being washed and followed over 3 days. In these experiments, we also observed a decrease in ZNF768 levels that was associated with a rise in the expression of senescence markers (Fig. 4C and Supplementary Fig. 4A). As previously reported^26^, Akt was activated in cells treated with doxorubicin and such activation was tightly linked to the degradation of ZNF768 (Supplementary Fig. 4B). Supporting the results presented above, we found that inhibiting Akt or blocking the proteasome were both sufficient to prevent doxorubicin-mediated ZNF768 degradation (Supplementary Fig. 4C, D).

### ZNF768 overexpression contributes to bypass RAS-induced senescence

To determine the role of ZNF768 downregulation during oncogene-induced senescence, rescue experiments were performed by overexpressing ZNF768 in primary mouse embryonic fibroblasts (MEFs) during RAS^G12V^-induced senescence. ZNF768 overexpression alone did not induce any obvious phenotype in MEFs. While the number of SA-β-gal positive cells increased significantly in response to oncogenic RAS^G12V^ expression, ectopic expression of ZNF768 was sufficient to block oncogene-induced senescence (Fig. 4D). To further explore the mechanism by which ZNF768 represses oncogene-induced senescence, IMR90 cells were transduced to overexpress RAS^G12V^, ZNF768, alone or in combination, together with shRNAs targeting either GFP or p53. As expected, RAS^G12V^ increased the number of SA-β-gal positive cells (Fig. 4E, lane 1 vs. 3), while ectopic expression of ZNF768 reduced the proportion of cells entering RAS-mediated senescence (Fig. 4E, lane 3 vs. 4). In a similar manner, p53 depletion also resulted in decreased amounts of SA-β-gal positive cells. However, combining ZNF768 overexpression and p53 depletion did not further reduce the number of SA-β-gal positive cells upon RAS^G12V^ expression (Fig. 4E, lane 4 vs. 6), suggesting that ZNF768 and p53 might act within the same tumor suppressor pathway. In addition, while RAS^G12V^-expressing cells are mostly undergoing a cell cycle arrest, expression of ZNF768 or depletion of p53 in RAS^G12V^-expressing allowed cell proliferation, as shown by BrdU incorporation assay (Supplementary Fig. 4E) and cell counting (Supplementary Fig. 4F). Further supporting the ability of ZNF768 to sustain proliferation in the context of RAS^G12V^ expression, we found that ZNF768 overexpression was associated with lower p21 and p53 and higher MCM6 protein levels (Supplementary Fig. 4G). Altogether, these results suggest that ZNF768 counteracts RAS^G12V^-induced senescence by negatively regulating the p53 tumor suppressor pathway.

### ZNF768 interacts with and represses p53 activity

**Fig. 5:**
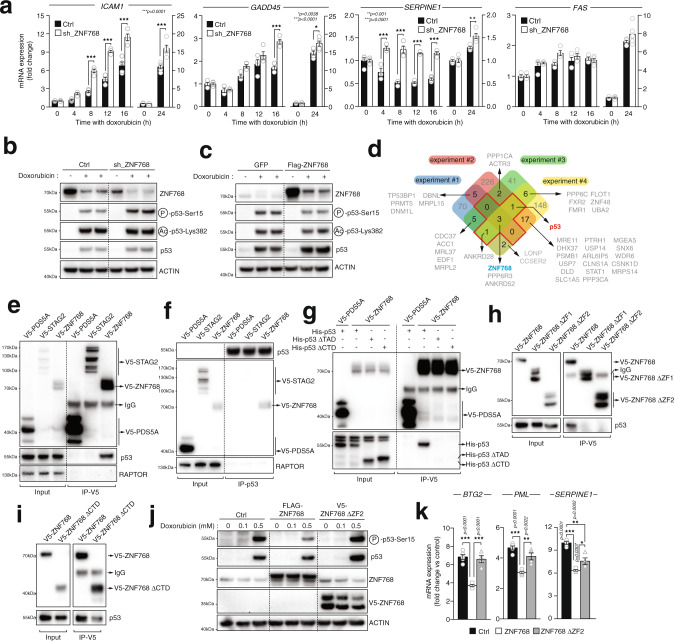
ZNF768 interacts with and represses p53 transcriptional activity. **A** U87 cells expressing a doxycycline-inducible shRNA targeting ZNF768 were treated or not with doxycycline (20 ng/µl) for 48 h. Cells were next treated with doxorubicin (0.5 µM). Gene expression was measured by RT-qPCR in at least three biological replicates per condition (*n* =  3/condition). Results are presented as fold change over controls (doxorubicin time 0). **B** Proteins were extracted from the cells described in (**A**) and western blots were performed. **C** U87 were transduced to overexpress Flag-ZNF768. Cells were treated for 24 h with doxorubicin (0.5 µM) and western blots were performed. **D** Venn diagram presenting the results of four independents rounds of immunoprecipitation (IP). 293T cells were transfected with V5-ACTA or V5-ZNF768 and V5-IPs were performed. Immunoprecipitates were analyzed by mass spectrometry. **E** 293T cells were transfected and V5 proteins were immunoprecipitated and western blots were performed. **F** 293T cells were transfected as described in (**E**) and endogenous p53 was immunoprecipitated and western blots were performed. **G** 293T cells were transfected with the indicated vectors. V5 proteins were immunoprecipitated and western blots were performed. **H**, **I** 293T cells were transfected with V5-ZNF768, V5-ZNF768ΔZincFinger (ZF)1 (missing amino acids 400 to 540), V5-ZNF768ΔZF2 (missing amino acids 252–540), or V5-ZNF768ΔCTD (missing amino acids 1–162). V5 proteins were immunoprecipitated and western blots were performed. **J** HCT116 cells were transduced to overexpress Flag-ZNF768 or V5-ZNF768ΔZF2. Cells were treated for 24 h with doxorubicin (0.5 µM). Proteins were extracted and western blots were performed. **K** RT-qPCR were performed from cells treated as described in (**J**) in at least four independent biological samples per condition (*n* = 4). In all panels, data represent the mean ± SEM. In panel **A**, significance was determined by two-way ANOVA with Tukey’s multiple-comparisons test. In panel **K**, significance was determined by one-way ANOVA with Tukey’s multiple-comparisons test. Details about reproducibility are provided in the Statistics and reproducibility included in the Methods section. Source data are provided as a Source data file.

In order to refine the relationship between ZNF768 and p53, U87 cells expressing a doxycycline-inducible shRNA targeting ZNF768 were acutely treated with doxycycline to knockdown ZNF768 and were next exposed to doxorubicin to activate p53 (Supplementary Fig. 5A, B). The expression of established p53 target genes was next assessed as a measure of p53 activity. As depicted in Fig. 5A, we observed a pronounced increase in the expression of numerous p53 target genes in cells depleted from ZNF768 and exposed to doxorubicin. In line with these findings, we also measured a rise in the expression of p53 targets when ZNF768-depleted cells were exposed to Nutlin3a, a small molecule that activates p53 (Supplementary Fig. 5C–E)^27^. Strikingly, ZNF768 overexpression repressed the expression of several p53 target genes in cells treated with Nutlin3a (Supplementary Fig. 5F). The repression in p53 target genes by ZNF768 overexpression was also reproduced in HCT116 cells, indicating that the control of p53 function by ZNF768 is conserved in various cell lines (Supplementary Fig. 5G, H). Taken together, these results indicate that ZNF768 negatively regulates p53 activity.

To understand how ZNF768 represses p53 activity, we looked at p53 total levels, acetylation and phosphorylation in some of the experiments described above, as these parameters are recognized to play key roles in controlling p53 function^28^. As shown in Fig. 5B, C, acute ZNF768 knockdown and overexpression minimally affected p53 total protein and p53 acetylation, but induced profound changes in p53 phosphorylation state. In details, an increase in the phosphorylation of p53 was observed in U87 cells depleted from ZNF768 and acutely treated with doxorubicin (Fig. 5B). These findings were reproduced in HCT116 cells (Supplementary Fig. 5I). Consistently, the opposite phenotype was observed in response to ZNF768 overexpression (Fig. 5C). Importantly, the modulation in p53 phosphorylation was not linked to variation in the activation of key kinases controlling the phosphorylation of these residues, indicating that ZNF768 unlikely represses p53 phosphorylation and activation through the regulation of these upstream regulators (Supplementary Fig. 5I). Supporting previous literature showing that p53 phosphorylation impacts p53 stability^29,30^, we found that ZNF768-mediated repression in p53 phosphorylation was associated with a reduction in total p53 levels several days after exposing cells to doxorubicin (Supplementary Fig. 5G). This effect of ZNF768 on p53 expression was also observed in the context of RAS-induced senescence (Supplementary Fig. 4G, lane 4 vs. lane 3)

In order to gain insights into the molecular mechanism linking ZNF768 to p53, we next performed immunoprecipitation (IP) and MS experiments to define the binding partners of ZNF768. Briefly, V5-tagged ZNF768 or V5-control proteins were overexpressed in 293T cells and independent rounds of IPs followed by MS analyses were performed. These experiments identified 42 proteins that were co-immunoprecipitated with ZNF768 in at least 2 of 4 IP–MS runs (Fig. 5D). Using this approach, p53 was identified as a potential ZNF768-binding partner. In follow-up experiments, we observed that V5-ZNF768 or endogenous ZNF768 were efficiently immunoprecipitating p53 (Fig. 5E and Supplementary Fig. 5J). Further confirming this interaction, we also observed that IP of either endogenous p53 or V5-p53 could immunoprecipitate ZNF768 (Fig. 5F and Supplementary Fig. 5K). We noticed that deleting the transactivation domain 1 and 2 (TAD1 and TAD2) of p53, a central hub for various co-factors and partners of p53, completely prevented the binding of p53 with ZNF768 (Fig. 5G and Supplementary Fig. 5L). Moreover, deletion of the C-terminal part of p53, that contains the oligomerization domain and a regulatory domain, two sections required for the tetramerization and the activation of p53, also impaired the interaction with ZNF768 (Fig. 5G and Supplementary Fig. 5L). These results indicate that the N-terminal part of p53 and its oligomerization are contributing to the interaction with ZNF768. Complementary experiments were next performed to define the domains of ZNF768 involved in the interaction with p53. In these experiments, we found that deleting the C2H2 domains in ZNF768 completely prevented the interaction with p53 (Fig. 5H and Supplementary Fig. 5M). On the other hand, deletion of the CTD domain containing the heptapeptide repeats did not prevent the binding of ZNF768 to endogenous p53 (Fig. 5I and Supplementary Fig. 5M), indicating that this region of ZNF768 does not mediate the interaction with p53. Altogether, the results presented above suggest that ZNF768 might repress p53 function by interfering with its phosphorylation through physical interaction. In order to test this possibility, cells were next transduced to overexpress either wild type ZNF768 or mutant isoforms that do not bind to p53 (ZNF768 ΔZF2) and the phosphorylation state of p53 and its activity were assessed, as described above. We found that preventing ZNF768 binding to p53 was sufficient to restore p53 phosphorylation in cells treated with doxorubicin (Fig. 5J). In these cells, the expression of p53 target genes was either partially or completely normalized compared to control cells (Fig. 5K). Overall, these results indicate that ZNF768 physically interacts with p53 to repress its phosphorylation and prevent its full activation.

### Elevated ZNF768 expression and protein levels in human cancers

**Fig. 6:**
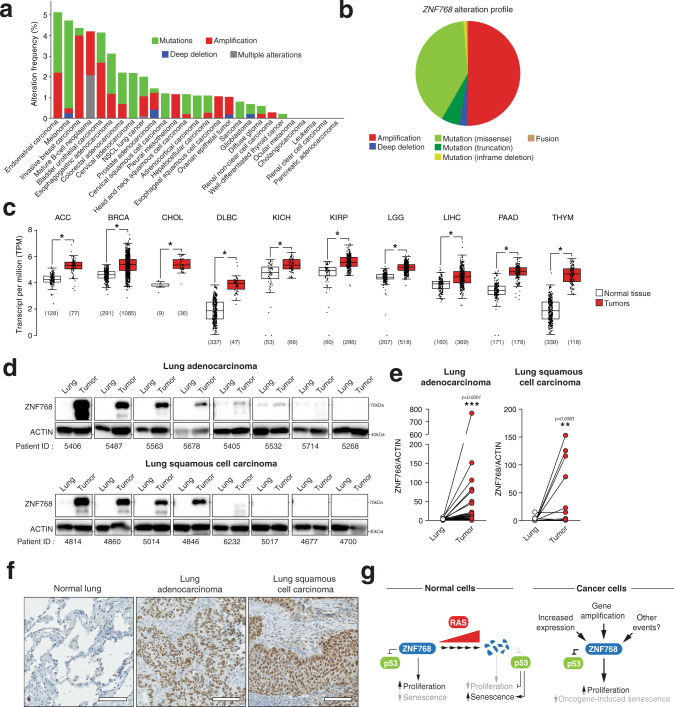
ZNF768 expression and protein levels are elevated in several human cancers. **A** Large-scale cancer genomics analysis of *ZNF768* mutation profile performed using cBioportal and TCGA PanCancer Atlas Studies. **B** Distribution of *ZNF768* alteration profile based on the analysis described in (**A**). **C** Gene expression analysis of ZNF768 expression between normal and cancer tissues. This analysis was performed using the GEPIA resource. The method for differential analysis is one-way ANOVA, without adjustment for multiple comparisons. The Log2 fold change cut-off was set up at 0.5 and the *p*-value cut-off at 0.001 (*p < 0.001). Each dot represents one sample. The number (*n*) of sample analyzed is provided below each graph bar. The low and high limits of the box represent the lower and upper quartiles, respectively. The middle line in the box represents the median value. The whiskers are 1.5 times the interquartile range. **D** Western blot analyses of ZNF768 protein levels in lung tumors (adenocarcinoma (*n* = 28) or squamous cell carcinoma (*n* = 10) or normal lung samples collected from human patients. Representative samples are shown from the same exposure time. **E** Quantification of ZNF768 protein levels in the experiment described in (**D**). Significance was determined on log transformed data using two-tailed paired *t* test. **F** Immunohistochemistry analyses of ZNF768 protein in normal lung and lung tumors. Examples of tumors showing high levels of ZNF768 are presented (Scale: 100 µm)**. G** Presentation of the proposed functions of ZNF768 in normal and cancer cells. In all panels, data represent the mean ± SEM. Details about reproducibility are provided in the Statistics and reproducibility included in the Methods section. Source data are provided as a Source data file.

Owing to the importance of ZNF768 in supporting cell proliferation, we next sought to define whether *ZNF768* gene is altered or its expression modulated in human cancers. Analyses of the TCGA PanCancer Atlas Studies through cBioPortal^31^ revealed that *ZNF768* gene is altered in 1.9% of tumors, with the highest incidence being observed in endometrial carcinomas (5.1%), melanoma (4.7%), invasive breast cancer carcinoma (4.3%), mature B-cell neoplasm (4.2%), and bladder urothelial carcinoma (4.1%) (Fig. 6A). A deeper analysis next showed that gene amplification is by far the most frequent alteration found in *ZNF768* gene (Fig. 6B). We found that *ZNF768* is amplified in 50.5% of all the tumor samples with altered *ZNF768*. These analyses also revealed that *ZNF768* loss is very uncommon in cancer (Fig. 6A, B). Indeed, the complete deletion of *ZNF768* was observed in only 0.05% of all the tumors included in the TCGA PanCancer Atlas Study. These findings support our previous results showing that severe ZNF768 depletion is not well tolerated by cancer cells in vitro. We next performed gene expression analyses using GEPIA, a web tool based on TCGA and GTEx data, to define whether *ZNF768* transcript levels are affected in human cancer^32^. This analysis revealed that *ZNF768* expression is significantly elevated in 10 out of the 31 cancer types analyzed (Fig. 6C and Supplementary Fig. 6A). High *ZNF768* expression was detected in adrenocortical carcinoma (ACC), breast invasive carcinoma (BRCA), cholangiocarcinoma (CHOL), diffuse large B-cell lymphoma (DLBC), kidney chromophobe (KICH), kidney renal papillary cell carcinoma (KIRP), lower grade glioma (LGG), liver hepatocellular carcinoma (LIHC) pancreatic adenocarcinoma (PAAD), and thymoma (THYM) (Fig. 6C). Only two cancer types showed lower *ZNF768* expression in tumors compared to normal tissue, namely acute myeloid leukemia (LAML) and testicular germ cell tumors (TGCT) (Supplementary Fig. 6A). Altogether, these results indicate that *ZNF768* is often amplified and/or overexpressed in various cancer types in humans.

Because ZNF768 is a protein strongly regulated at the post-translational level, we have initiated studies to measure ZNF768 protein levels in human tumors. Here, we took advantage of samples collected from patients with lung adenocarcinoma (LUAD) or lung squamous cell carcinoma (LUSC), the most common forms of non-small cell lung carcinomas. These cancer types were chosen for two reasons. First, LUAD and LUSC often carry oncogenic mutations in RAS proteins^33^. Second, these cancer types do not show severe changes in *ZNF768* mRNA levels (Supplementary Fig. 6A). As shown in Fig. 6D, E, we found that many lung tumors showed elevated ZNF768 protein expression relative to the normal adjacent lung tissue. Immunohistochemistry (IHC) assays revealed a strong nuclear staining in cancer cells of both LUAD and LUSC (Fig. 6F). Strikingly, high ZNF768 protein levels were measured in many of the lung tumors carrying oncogenic RAS^G12V^ mutations (Supplementary Fig. 6B), indicating a disconnection between RAS activation and ZNF768 degradation in advanced tumors. In order to define whether ZNF768 protein levels are also elevated in other cancer types, we looked at publicly available IHC data available through the Human Protein Atlas resource (www.proteinatlas.org)^34^. Many cancer types, including colorectal, breast and prostate adenocarcinomas, also displayed strong nuclear staining for ZNF768 (Supplementary Fig. 6C). These results indicate that high ZNF768 may be a common feature of various tumors in humans.

Studies in cancer cells in vitro showed that alteration in ZNF768 levels deeply impacts gene expression (Fig. 3). In order to define whether ZNF768 also affects gene expression profiles in human cancer, we used TCGA data and performed pathway analyses comparing lung tumors with either low or high *ZNF768* mRNA expression based on the median for each histological subtypes. The GSEA analysis for the LUAD (*n* = 533) and LUSC (*n* = 502) tumor samples resulted in 55 and 58 statistically significant pathways respectively (false-discovery rate (FDR) < 1%) (Supplementary Tables 1 and 2). Of these, 10 and 9 pathways were upregulated in the high ZNF768 group compared to the low ZNF768 group among the LUAD and LUSC patients respectively. More importantly, we found an enrichment of the cell cycle pathway in tumors with high *ZNF768* expression (Supplementary Tables 1 and 2). This effect was observed in both LUAD and LUSC samples. These results are consistent with our previous data showing the importance of ZNF768 for the expression of several cell cycle genes. Although additional studies are needed to define the precise contribution of ZNF768 to human cancer, these observations suggest that cells might use ZNF768 to sustain proliferation, bypass senescence and promote malignant transformation.

## Discussion

Although RAS signaling is important to support proliferation and promote cancer development, oncogenic activation of RAS in primary cells triggers cellular senescence^8^. The precise mechanisms by which pre-neoplastic cells support hyperactive RAS to bypass cellular senescence are still incompletely characterized. Here, we report the identification of ZNF768 as a protein linking oncogenic RAS to cellular senescence. Our results show that ZNF768 is destabilized upon RAS activation. We show that ZNF768 depletion impairs proliferation and induces senescence by modulating the expression of key cell cycle effectors and established p53 targets. We show that ZNF768 levels decrease in response to replicative-, stress- and oncogene-induced senescence, and that ZNF768 overexpression contributes to bypass RAS-induced senescence, in a p53-dependent manner. We provide evidence that ZNF768 interacts with and represses the phosphorylation and the activity of p53. Human cancer studies revealed that ZNF768 is often overexpressed in tumors, suggesting that cells might use this protein to bypass senescence, sustain proliferation and promote malignant transformation.

ZNF768 is the only zinc finger protein that carries amino acids stretches resembling the heptapeptide repeats found in the CTD of RPB1. Compared to RPB1, which contains 52 consensus motifs (Y_1_S_2_P_3_T_4_S_5_P_6_S_7_), ZNF768 carries between 9 and 19 degenerated repeats, depending on the homology threshold considered. In RPB1, these repeats are phosphorylated to regulate transcriptional processes^35^. The dynamic phosphorylation of the CTD, often defined as the “CTD code”, allows RPB1 to integrate inputs from several sources to control transcription^21^. Here, we show that ZNF768 is phosphorylated on the heptapeptide repeats in response to RAS activation, an effect that primes its degradation by the proteasome. Mutations of several serine residues embedded in these repeats increased ZNF768 levels. We also found that inhibition of both PI3K and MAPK signaling pathways additively increased ZNF768 stability. Our findings suggest that the heptapeptide repeats in ZNF768 may serve as a signaling hub allowing cells to integrate RAS activity to control cell proliferation. Whether specific phosphorylation signatures, or “CTD code”, control ZNF768 functions is an attractive possibility that warrants further investigation.

The complete depletion of ZNF768 represses proliferation. We found that severe ZNF768 loss causes cell cycle defects and promotes senescence and apoptosis in many cell types. Additional studies using a doxycycline-inducible approach revealed that the partial loss of ZNF768 also reduces proliferation. However, in that case, this effect was linked to a rise in cellular senescence but no increase in apoptosis. These findings indicate that ZNF768 is essential to sustain proliferation and that partial ZNF768 loss is sufficient to trigger cellular senescence. Supporting the link between ZNF768 and cellular senescence, we showed that ZNF768 is rapidly depleted during replicative-, stress- and oncogene-induced senescence and that its overexpression contributes to bypass this process. The rapid degradation of proteins required for proliferation is a common process that takes place during cellular senescence^13^. Our findings show that ZNF768 is likely part of this senescence-associated protein degradation program and suggest that ZNF768 could represent a novel functional marker of cellular senescence.

ZNF768 is a transcription factor that binds to MIR sequences^23^, a group of retrotransposed DNA elements associated with transcriptionally active euchromatin^36,37^. In addition to MIR, ZNF768 also interacts with the promoter of various genes to control their expression^23^. Overexpression of dominant negative ZNF768 revealed that ZNF768 controls the expression of several genes, at least in part in a cell type-specific manner^23^. In U2OS cells, many genes regulated by ZNF768 were annotated as DNA binding or zinc finger-containing genes, indicating that ZNF768 is hierarchically located upstream of a broad network of transcription factor genes^23^. A closer look at the data presented in this report also shows that repressing ZNF768 reduces the expression of various genes playing roles in cell cycle, cell division and mitosis. Here, we analyzed gene expression upon ZNF768 depletion in several cell lines and confirmed the cell-specific impact of ZNF768 on gene expression. Importantly, ontology analyses of the genes affected by ZNF768 in more than one line also revealed a strong enrichment for genes linked to the cell cycle and mitosis. Many genes identified by Rohrmoser et al.^23^ to be repressed upon ZNF768 depletion were confirmed in our study (Supplementary Table 3). These results indicate that, although ZNF768 controls the expression of genes in a cell-type specific manner, this transcription factor also impacts the expression of a core group of genes that support proliferation. Since ZNF768 is upstream of several other transcription factors, we cannot rule out that part of the effects of ZNF768 depletion on gene expression might be indirect. Regardless of its direct or indirect impact on gene expression, we provide clear evidence that ZNF768 is a key protein relaying growth factor signaling to the control of proliferation.

In addition to impact the expression of pro-proliferative genes, we found that ZNF768 also affects the expression of many *bona fide* p53 targets. In ZNF768-depleted cells, the rise in the expression of p53 targets occurred after the reduction in the expression of cell cycle related genes, suggesting that ZNF768 may act through independent but complementary mechanisms to regulate cell proliferation. On one hand, ZNF768 controls a gene network important for cell cycle progression, mitosis and cell division. On the other hand, ZNF768 physically interacts with p53 and its depletion amplifies p53 phosphorylation and activity. These p53-dependent and -independent modes of action likely explain why ZNF768 depletion impairs proliferation in both p53^+/+^ and p53^−/−^ cells. Although ZNF768 loss decreased proliferation irrespectively of the p53 status, senescence features were significantly increased in p53^+/+^ cells, indicating a role for p53 in promoting cellular senescence in ZNF768-depleted cells. Consistently, we observed that ZNF768 overexpression contributed to bypass cellular senescence in primary cells, and that this effect was dependent on p53. Altogether, our findings indicate that ZNF768 is a key player in determining the balance between cellular senescence and proliferation. We propose that the degradation of ZNF768 upon oncogenic RAS activation could thus represent a safeguard mechanism to promote cellular senescence, repress proliferation and protect against malignant transformation.

High ZNF768 protein levels were found in nearly half of the lung tumor samples tested. Because *ZNF768* is rarely amplified or overexpressed in lung tumors, these results indicate that post-transcriptional and/or post-translational processes likely take place to enforce ZNF768 protein expression in cancer. Thus, elevated ZNF768 levels might be more common in tumors than predicted from cancer genomics and transcriptomics analyses. Supporting this possibility, analysis of ZNF768 protein levels through the Human Protein Atlas revealed that many human cancers display intense staining for ZNF768. In line with these observations, previous reports showed that autoantibodies against ZNF768 are detected in the plasma of patients with colorectal cancer^38,39^, a cancer type in which *ZNF768* mRNA levels are not increased. Our findings suggest that sustained ZNF768 expression might offer a proliferative advantage to cancer cells by promoting the expression of key cell cycle regulator and by repressing p53 activity and cellular senescence. Additional studies will be needed to define the role of ZNF768 in human cancer.

In conclusion, we provide evidence that ZNF768 is a downstream effector of RAS that serves as a central checkpoint to couple growth factor signaling to the control of proliferation. We propose a model in which oncogenic RAS promotes ZNF768 degradation, which represses the expression of key proliferative genes, amplifies p53 activity and triggers cellular senescence (Fig. 6G). This safeguard mechanism against neoplasia is bypassed by cancer cells, that often overexpress ZNF768 (Fig. 6G).

## Methods

### Cell culture and reagents

All the cell lines (RPE1, Hela, U87, HCT116, MDAMB231, HEK 293, Caco-2, HT29, IMR90) were obtained from American Type Culture Collection (ATCC) or Coriell Institute and cultured according to standard mammalian tissue culture protocols and sterile technique. The cell lines were cultured in complete Dulbecco’s Modified Eagle Medium (DMEM) supplemented with Fetal Bovine Serum (FBS) (10%) (Sigma, #F1051) and penicillin-Streptomycin (1%) (Wisent, #450-201-EL). The following reagents were used in cell culture experiments: Tamoxifen (Sigma, #T5648), PD098059 (Cayman Chemical, #10006726), Torin1 (Cayman Chemical, #10997), Cycloheximide (Sigma, #C7698), Puromycin (Sigma, #P8833), Blasticidin S-HCl (ThermoFischer Scientific, #A1113903), Hygromycin B (BioShop Canada, #HYG002), Akt inhibitor VIII (Cayman Chemical #14870), MG132 (Cayman Chemical, #10012628), Doxycycline (Sigma, #D3447), Doxorubicin (Tocris, #2252), Nutlin3a (Cayman Chemical, #18585) and Rapamycin (LC Laboratories, #R5000).

### Virus production and infection

Retroviruses were produced using gag/pol and CMV VSV-G as the packaging system. Lentiviruses were produced using psPAX2 and pMD2G. 293T cells were transfected with the vectors. Virus-containing supernatants were collected at 48 h after transfection and filtered using a 0.45μm filter. Cells were transduced for 24 h in the presence of 8 μg/ml polybrene. After infection, the cells were dispersed into fresh medium. Cells were selected on the following days with either 1 μg/mL puromycin or with 2.5 μg/mL of blasticidin, depending on the viral constructs.

### Vectors

Lentiviral shRNAs were obtained from the collection of The RNAi Consortium (TRC) at the Broad Institute. These shRNAs are named with the numbers found at the TRC public website: shAKT1_1 (TRCN0000010174), shAKT1_2 (TRCN0000010171), shAKT2_1 (TRCN0000255915), shAKT2_2 (TRCN0000255917), shRAPTOR_1 (TRCN0000010415), shRAPTOR_2 (TRCN0000039772), shRICTOR_1 (TRCN0000074290), shRICTOR_2 (TRCN0000074291), shZNF768_1 (TRCN0000017384), shZNF768_2 (TRCN0000017385). For the inducible depletion of ZNF768, the hairpin sequence of shZNF768_1 (TRCN0000017384) was cloned in Tet-pLKO-puro (gift from Dmitri Wiederschain, Addgene plasmid # 21915). Lentiviral constructs for overexpression of V5-tagged proteins were obtained from the collection CCSB Broad Resource. The sequence of these vectors can be found at the TRC public website: pLX304_V5-ACTA1 (ccsbBroad304_13807), pLX304_V5-PDS5A [transcript variant 3] (ccsbBroad304_02741), pLX304_V5-STAG2 (ccsbBroad304_02516), pLX304_V5-TP53 (ccsbBroad304_07088) and pLX304_V5-ZNF768 (ccsbBroad304_12602). The V5-ZNF768 S to A phosphomutants were generated using the QuickChange Site-Directed Mutagenesis kit (Agilent, 200519). pLX304_V5-ZNF768 was used as template for the mutagenesis protocol. ZNF768, FLAG-ZNF768, V5-ZNF768ΔZF1, V5-ZNF768ΔZF2, V5-ZNF768ΔCTD were also subcloned in MSCV (Addgene plasmid # 24828, a kind gift from Dr. Lin He) using pLX304_V5-ZNF768 as template. ZNF768 was subcloned into pRCF (kind provided by Dr. Jacques Côté) using pLX304_V5-ZNF768 as template. pBabe-Puro-Myr-Flag-Akt1 (Myr-Akt) was a gift from William Hahn (Addgene plasmid #15294). pLNCX2 ER:ras (ER1a-HRAS^G12V^) was a gift from Masashi Narita (Addgene plasmid #67844). pRS sh_GFP and pRS sh_p53 were obtained from Dr. Reuven Agami^40^. pWZL Hygro-H-Ras^G12V^ was a gift from Dr. Scott Lowe (Addgene plasmid #18749) and pWZL blast-H-RAS^G12V^ was provided by Dr. Masashi Narita. His-p53 and His-p53 Δ-TAD were subcloned in MSCV using pGEX-human p53-(1-393) as a template (gift from Cheryl Arrowsmith, Addgene plasmids #24860). His-p53 Δ-CTD was subcloned in MSCV using pGEX-human p53 (1-320) as a template (gift from Cheryl Arrowsmith, Addgene plasmids #24864).

### Protein stability experiments

Cells were treated with cycloheximide (50 µM) and cells were lysed at the indicated times. ZNF768 stability was measured by comparing the protein levels 1, 2, 4, and 8 h to the untreated cells.

### Immunoprecipitations

Cells were rinsed twice with ice-cold PBS. Cells were next lysed in a buffer containing 40 mM HEPES [pH 7.4], 2 mM ethylenediaminetetraacetic acid [EDTA], 10 mM sodium pyrophosphate, 10 mM sodium glycerophosphate, 150 mM NaCl, 50 mM NaF, 1% Triton X-100, and one table of EDTA-free protease inhibitors/25 ml of buffer. The soluble fractions of cell lysates were isolated by centrifugation at 16,000 g for 10 min in a microcentrifuge. For IPs, primary antibodies were added to the lysates and incubated with rotation for 4 h at 4 °C. The following primary antibodies were used (V5-Tag [D3H8Q, Cell Signaling Technology, #13202], p53 [Cell Signaling Technology, #2524], ZNF768 [Aviva Systems Biology, FLJ23436], Phospho-MAPK/CDK substrates (PXS*P or S*PXR/K) [Cell Signaling Technology, #2325]). A 50% slurry of protein G Sepharose was then added, and the incubation continued overnight. Immunoprecipitated proteins were extensively washed before being either denatured and analyzed by western blot, or used for MS analyses, as described above.

### Western blotting

All cells were rinsed twice with ice-cold phosphate-buffered saline (PBS) before lysis. Cells were lysed with Triton-X 100 containing lysis buffer (50 mM HEPES, pH 7.4, 2 mM EDTA, 10 mM sodium pyrophosphate, 10 mM sodium glycerophosphate, 40 mM NaCl, 50 mM NaF, 2 mM sodium orthovanadate, 1% Triton-X 100, and one tablet of EDTA-free protease inhibitors per 25 ml). Tissues were homogenized with the same buffer supplemented with 0.1% sodium lauryl sulfate and 1% sodium deoxycholate. Cells and tissues were rotated at 4 °C for 10 min and then the soluble fractions of cell lysates were isolated by centrifugation for 10 min in a microcentrifuge. Protein levels were then quantified using Bradford reagent and analyzed by Western blotting. Protein extracts were diluted in sample buffer, denaturated by heat (95 °C) for 10 min and loaded on precast gels (Life Technologies). Proteins were transferred to PVDF membranes blocked in 5% milk diluted in PBS-Tween and incubated with their primary antibody overnight at 4 °C. The following antibodies were used: ZNF768 [Aviva Systems Biology, FLJ23436, dilution 1 :1000]; Akt (pan) [C67E7, Cell Signaling Technology, #4691, dilution 1:1000]; Phospho-Akt (ser473) [D9E, Cell Signaling Technology, #9271, dilution 1:1000], Phospho-Akt (Thr308) [244F9, Cell Signaling Technology, #9275, dilution 1:1000], p44/42 MAPK (Erk1/2) [137F5, Cell Signaling Technology, #9102, dilution 1:1000], Phospho-p44/42 MAPK (Erk1/2)(Thr202/Tyr204)[D13.14.4E, Cell Signaling Technology, #9101, dilution 1:1000], p70 S6 kinase [Cell Signaling Technology, #9202, dilution 1:1000], Phospho-p70 S6 Kinase (Thr389) [Cell Signaling Technology, #9205, dilution 1:1000], Raptor [24C12, Cell Signaling Technology, #2280, dilution 1:1000], Rictor [53A2, Cell Signaling Technology, #2114, dilution 1:1000], V5-Tag [D3H8Q, Cell Signaling Technology, #13202, dilution 1:1000], Caspase-3 [Cell Signaling Technology, #9662, dilution 1:1000], PARP [46D11, Cell Signaling Technology, #9532, dilution 1:1000], p21 Waf1/Cip1 [12D1, Cell Signaling Technology, #2947, dilution 1:1000], p16 INK4A [D7C1M, Cell Signaling Technology, #80772, dilution 1:1000], p53 [1C12, Cell Signaling Technology, #2524, dilution 1:1000], Phospho-p53 (Ser15) [16G8, Cell Signaling Technology, #9286, dilution 1:1000], Phospho-p53 (Ser33) [Cell Signaling Technology, #2526, dilution 1:1000], Acetyl-p53 (Lys382) [Cell Signaling Technology, #2525, dilution 1:1000], Cyclin D1 [92G2, Cell Signaling Technology, #2978, dilution 1:1000], Phospho-histone H3 (Ser10) [D2C8, Cell Signaling Technology, #3377, dilution 1:1000], Phospho-histone H2A.X (Ser139) [20E3, Cell Signaling Technology, #9718, dilution 1:1000], S6 Ribosomal Protein [5G10, Cell Signaling Technology, #2217, dilution 1:2500] Cyclin B1 [D5C10, Cell Signaling Technology, #12231, dilution 1:1000], β-actin [Cell Signaling Technology, #4967, dilution 1:1000], Phospho-ATM (ser1981) [Cell Signaling Technology, #5883, dilution 1:1000], Phospho-CHK1 (Ser345) [Cell Signaling Technology, #2348, dilution 1:1000], Phospho-CHK2 (Thr68) [Cell Signaling Technology, #2197, dilution 1:1000] FLAG [M2, Sigma Aldrich, F3165, dilution 1:1000], MCM6 [Bethyl Laboratories, # A300-194A, 1:2000], phospho-Rb (T826) [EPR5351, Abcam, # ab133446, dilution 1:1000], α-Tubulin [B-5-1-2, Sigma Aldrich, #T5168, dilution 1:20000], p21 [C-19, Santa Cruz Biotechnologies, # SC397, dilution 1:500], p53 HRP-Conjugated [R&D Systems, HAF1355, dilution 1:5000]. Secondary antibodies were purchased for Cell Signaling Technology [Cell Signaling Technology, #7074, #7075, dilution 1:5000]. ChemiDoc MP Imager and ChemiDoc and Image Lab software (version 5.1) were used to acquire and analyze images.

### MS analyses

Proteins on beads were washed 3 times with 50 mM ammonium bicarbonate buffer and digested with trypsin (1 µg) overnight at 37 °C. Reaction was stopped by acidification with 3% acetonitrile–1% TFA–0.5% acetic acid. Beads were removed and the peptides were purified on stage tip (C18) and vacuum dried before MS injection. Samples were solubilised into 10 µl of 0.1% formic acid and by MS. Peptide samples were analyzed by LC-MS/MS using an Ekspert NanoLC425 (Eksigent) coupled to a 5600+ mass spectrometer (Sciex, Framingham, MA, USA) equipped with a nanoelectrospray ion source. Peptides were trapped at 4 μl/min in loading solvent (0.1% formic acid) on a 5 mm×300 μm C18 PepMap cartridge pre-column. Peptides were eluted with a linear gradient from 5 to 35% solvent B (acetonitrile, 0.1% formic acid) in 35 min, at 300 nL/min. Mass spectra were acquired using a data dependent acquisition mode using Analyst software version 1.7. Each full scan mass spectrum (400 to 1250 m/z) was followed by collision-induced dissociation of the twenty most intense ions. Dynamic exclusion was set for a period of 12 sec and a mass tolerance of 100 ppm. MGF peak list files were created using Protein Pilot version 4.5 software (Sciex). MGF sample files were then analyzed using Mascot (Matrix Science, London, UK; version 2.5.1). Mascot was set up to search the Uniprot complete proteome HomoSapiens database (https://www.uniprot.org/) and a contaminant database assuming the digestion enzyme trypsin. Mascot was searched with a fragment ion mass tolerance of 0,1 Da and a parent ion tolerance of 0.1 Da. Scaffold (version Scaffold_4.8.4, Proteome Software Inc., Portland, OR) was used to validate MS/MS based peptide and protein identification. Protein probabilities were assigned by the Protein Prophet algorithm^41^. Peptides and Proteins were validated if their FDR were less than 1%. Proteins that contained similar peptides and could not be differentiated based on MS/MS analysis alone were grouped to satisfy the principles of parsimony.

### SA-β-gal activity assay

Senescence-associated β-galactosidase (SA-β-gal) assays were performed as previously described^42,43^. Cells were fixed with 0.5% glutaraldehyde in PBS for 15 min, then washed and kept in PBS (pH 5.5 for MEF or pH 6.0 for IMR90) containing 1 mM of MgCl_2_, for at least 24 h. Staining was performed at 37 °C using a solution containing X-Gal, potassium ferricyanide, potassium ferrocyanide and MgCl_2_ in PBS (pH 5.5 or pH 6.0). Images were taken and the percentage of SA-β-gal positive cells was quantified.

### BrdU incorporation

IMR90 cells were grown on coverslips and incubated for 6 h with 0.1 mg/mL 5-Bromo-2′-Deoxyuridine (BrdU, VWR, # B5002) prior to fixation in 4% paraformaldehyde in PBS for 15 min. Cells were then incubated for 20 min in permeabilization buffer (0.1% Triton X-100 in PBS), followed by 10 min incubation in 1 N HCl, 10 min in 2 N HCl and 10 min incubation in phosphate/citric acid buffer, pH 7.4. After three rounds of washes with permeabilization buffer, cells were incubated overnight with Alexa 488-conjugated anti-BrdU antibody (ThermoFisher Scientific B35130, 1:200), followed by washes and staining with 4,6-diamidino-2-phenylindole (DAPI). Images were captured using a Zeiss AxioObserver Z1 motorized inverted microscope. The percentage of BrdU incorporation was determined by counting at least 300 cells per condition using Image J software.

### Microarray analyses

Whole-genome gene expression was performed using the Affymetrix GeneChip Human Gene 2.0 ST Array. The RNA was labeled and hybridized using a standard Affymetrix protocol. The quality of arrays was judged using standard quality control parameters and all arrays passed the quality control filters. Expression values were extracted using the Robust Multichip Average method^44^ implemented in the *oligo* package in R^45^.

### Quantitative real-time PCR

Total mRNA was isolated from cells and tissues using the RNeasy Lipid Tissue Mini Kit (Qiagen, 74104). Total mRNA was isolated from cells using E.Z.N.A. Total RNA Kit I (Omega Biotek, R6834-02). RNA concentration was estimated from absorbance at 260 nm. cDNA synthesis was performed using the iScript^™^ Advanced cDNA Synthesis Kit for RT-qPCR (Bio-Rad). mRNA extraction and cDNA synthesis were performed following the manufacturer’s instructions. cDNA was diluted in DNase-free water (1:15) before quantification by real-time PCR. mRNA transcript levels were measured in duplicate samples using CFX96 or CFX384 touch^™^ real-time PCR (Bio-Rad, Mississauga, ON, Canada). Chemical detection of the PCR products was achieved with SYBR Green (Bio-Rad, 172-5271). At the end of each run, melt curve analyses were performed, and representative samples of each experimental group were run on agarose gel to ensure the specificity of amplification. Gene expression was corrected for the expression level of reference gene. The primer sequences used are presented in Supplementary Table S4.

### Live cell imaging

HeLa cells stably expressing GFP-tagged histone H2B (H2B–GFP cells) were plated onto 35-mm dishes and transduced with the respective shRNA. Live-cell imaging was performed with the TE 2000 microscope (Nikon) using a Photometrics CoolSnap HQ camera. Cell culture dishes were placed onto the microscope with an environmental control chamber, maintaining the temperature at 37 °C and the CO2 at 5%. The cells were imaged every 2 min for 16 h with a Prior motorized stage for four dishes, and a stack of images with a Z-step size of 4.5 µm was collected. All images were collected with Plan Fluor 40×lens (numerical aperture  =  0.6) with a Chroma filter set 41001 configured for EGFP fluorescent protein tags [i.e., a set of filters for excitation atl (emission)  =  488 nm and l (emission)  =  500–550 nm]. Exposure times were 450 ms with a camera binning of 2. With a scanning stage, Z-stacks (three steps of 1.5 µm) of approximately 6–10 cells were imaged at five different XY positions on three dishes during each experiment. The data were analyzed with MetaMorph software (Molecular Devices, version 7.8.4.0). Complementary experiments were performed wild-type Hela cells to analyze the mitotic defects linked to ZNF768 depletion. Following ZNF768 knockdown, cells were stained with 100 nm SiR-DNA and directly used in live-imaging microscopy for 8 h were imaged by confocal microscopy on an inverted Olympus IX80 microscope equipped with a WaveFX-Borealin-SC Yokagawa spinning disc (Quorum Technologies), a motorized stage (ASI) and an incubator with atmospheric CO_2_ heated to 37 °C and an Orca Flash4.0 camera (Hamamatsu). Image acquisition was performed using Metamorph software (Molecular Devices). Mitotic events, metaphase defects (alignments defects or no metaphase phenotype) and anaphase defects (lagging chromosomes and chromosome bridges) were counted.

### Human lung samples

The patients included in this study were diagnosed with either LUAD or LUSC and underwent surgical resection at the Institut universitaire de cardiologie et de pneumologie de Québec—Université Laval (IUCPQ-UL). Lung tumors and adjacent normal lung were collected and stored at the IUCPQ-UL site of the Respiratory Health Network Tissue Bank (www.tissuebank.ca). The Research Ethics Committee of IUCPQ-UL approved this study (#2017-2829, 21441) and all participants provided written and informed consent.

### Immunohistochemistry

IHC was performed on formalin-fixed paraffin-embedded tissue sections of 4 microns using charged slides. Slides were stained using a fully automated procedure on Dako Autostainer Link 48 (Dako-Agilent Technology, Santa Clara, CA) following heat induced epitope retrieval in a Dako P-T Link with citrate buffer pH: 6. IHC was carried out using EnVision FLEX visualization system (Dako). Endogenous peroxydase was inhibited by treating sections with FLEX 0.3% hydrogen peroxide for 5 min. Slides were next incubated for 20 min with polyclonal rabbit ZNF768 primary antibody (Aviva Systems Biology) at dilution 1:100. Flex HRP polymer was applied for another 20 min followed by visualization in DAB, and counterstaining in hematoxylin. Slides were cleared, covered and then scanned on Hamamatsu Nanozoomer 2.0 HT.

### Tumor sequencing

DNA was extracted from the tumors using a commercial kit (QIAGEN, #69504). KRAS G12 mutation status was analyzed by ddPCR using a the KRAS Screening Multiplex kit (Biorad, #18603506).

### Pathway enrichment analysis

The pathway enrichment analysis was performed by employing the gene set enrichment analysis (GSEA) method using the statistic obtained from the *t* test (that assesses the difference in the gene expression between the two groups). The enrichment score for each pathway was computed using the GSEA method with statistical significance calculated using a permutation test (10,000 permutations). Nominal *P*-values obtained for each pathway was corrected for multiple testing using the FDR approach, and a threshold of *P* < 0.01 was considered statistically significant.

### Statistics and reproducibility

All the statistical analyses were performed using Prism version 6.0.

*Figure* *1*: The results presented in Fig. 1B are representative of an experiment that was performed twice and in different cell lines. The results presented in Fig. 1C are representative of experiments that were performed at least two times. The results presented in Fig. 1D were reproduced in a section of Fig. 1H and were also reproduced in two other cell lines (see Supplementary Fig. 1F). The results presented in Fig. 1E were reproduced in a section of Fig. 1H. This experiment was also reproduced in four other cell lines (see Supplementary Fig. 1G). The results presented in Fig. 1F represent the average of three independent experiments (*n* = 3). The results presented in Fig. 1G represent the average of four independent experiments (*n* = 4). The results presented in Fig. 1H are representative of an experiment that was performed twice. The results presented in Fig. 1I, J were confirmed in Fig. 1K. The results presented in Fig. 1K were produced from at least six independent biological replicates per conditions (*n* = 6). The results presented in Fig. 1L were confirmed in Fig. 1M and were reproduced in another cell line. The results presented in Fig. 1M represent the average of two independent experiments (*n* = 2). The results presented in Fig. 1N were reproduced in a section of Fig. 1O. This experiment was also reproduced in another cell line (see Supplementary Fig. 1K). The results presented in Fig.1R are representative of an experiment that was performed at least twice with the same outcome. The result presented in Fig. 1S is representative of three independent experiments. The quantification is presented in Fig. 1T. The experiment presented in Fig. 1U was reproduced twice.

*Figure* *2*: The results presented in Fig. 2A were produced from independent studies performed in nine different cell lines. The results presented in Fig. 2B represent the average of three independent experiments (*n* = 3) performed in two different cell lines. The results presented in Fig. 2C are representative of another experiment performed in similar conditions. The results presented in Fig. 2D, E come from the analysis of three independent replicates (*n* = 3). The results presented in Fig. 2F come from an experiment that was performed once. The results presented in Fig. 2G were produced from independent studies performed in four different cell lines. Some of these results were also reproduced in Supplementary Fig. 2H, N. The results presented in Fig. 2H represent the average of two independent experiments for the U87 cells (*n* = 2) and three independent experiments for the Hela cells. These observations were also reproduced in another cell line (see Supplementary Fig. 2I). The results presented in Fig. 2I come from the analysis of six independent biological replicates per condition (*n* = 6). Similar results were also observed in other experiments (see Fig. 2E and Supplementary Fig. 2P).

*Figure* *3*: The results presented in Fig. 3C, D were produced from three independent biological replicates per condition (*n* = 3). The results presented in Fig. 3E come from the analysis of three independent samples per condition (*n* = 3). The results presented in this figure confirm findings presented in several panels, including Supplementary Fig. 3B.

*Figure* *4*: The results presented in Fig. 4A are representative of an experiment that was performed twice. The results presented in Fig. 4B are representative of an experiment that was performed twice. The results presented in Fig. 4C are  representative of an experiment that was reproduced in another cell line (see Supplementary Fig. 4A). The results presented in Fig. 4D represent  the average of four independent experiments (*n* = 4). The results presented in 4E represent  the average of three independent experiments (*n* = 3).

*Figure* *5*: The results presented in Fig. 5A come from the analysis of at least three independent biological samples per condition (*n* = 3). The results presented in Fig. 5B are representative of an experiment that was reproduced twice and in another cell line (see Supplementary Fig. 5I). The results presented in Fig. 5C are representative of an experiment that was reproduced in another cell line (see Fig. 5J). The results presented in Fig. 5D come from the analysis of four independent experiments (*n* = 4). The results presented in Fig. 5E–I are representative of experiments that were reproduced at least twice. Some of these results were also reproduced in Supplementary Fig. 5J, K. The results presented in Fig. 5J are representative of experiments that were reproduced at least twice. The experiment presented in Fig. 5K comes from the analysis of at least four independent biological replicates per condition (*n* = 4).

*Figure* *6*: The results presented in Fig. 6D, E come from the analysis of 38 human tumors and corresponding normal lungs (*n* = 28 LUAD and *n* = 10 LUSC). The results presented in Fig. 6F are part of a tissue microarray that was performed twice, with similar outcome for many lung tumors.

### Reporting summary

Further information on research design is available in the Nature Research Reporting Summary linked to this article.

## Supplementary information

Supplementary Information

Description of Additional Supplementary Files

Supplementary Data 1

Supplementary Data 2

Supplementary Data 3

Supplementary Data 4

Supplementary Data 5

Reporting Summary

## Data Availability

The source data underlying the all Figures and Supplemental Figures are included as Source Data Files. All data are available. The raw mass spectrometry data have been deposited to the ProteomeXchange Consortium via the PRIDE with the dataset identifier PXD027312 and the Uniprot Homo Sapiens proteome used in mass spectrometry analyses can be found at this link https://www.uniprot.org/. The microarray data have been deposited on GEO with the following accession code GSE176578. Source data are provided with this paper.

## Source data

Source Data
