# Supplementary material for: ZNF768 links oncogenic RAS to cellular senescence
Source: Nat Commun. 2021 Aug 17;12:4841. doi: 10.1038/s41467-021-24932-w (PMC8370976; doi:10.1038/s41467-021-24932-w)
Supplement: Supplementary file 1 — Supplementary Information [file 41467_2021_24932_MOESM1_ESM.pdf]

Supplementary Figure 1. Identification of ZNF768 as a new phosphoprotein destabilized upon RAS activation.

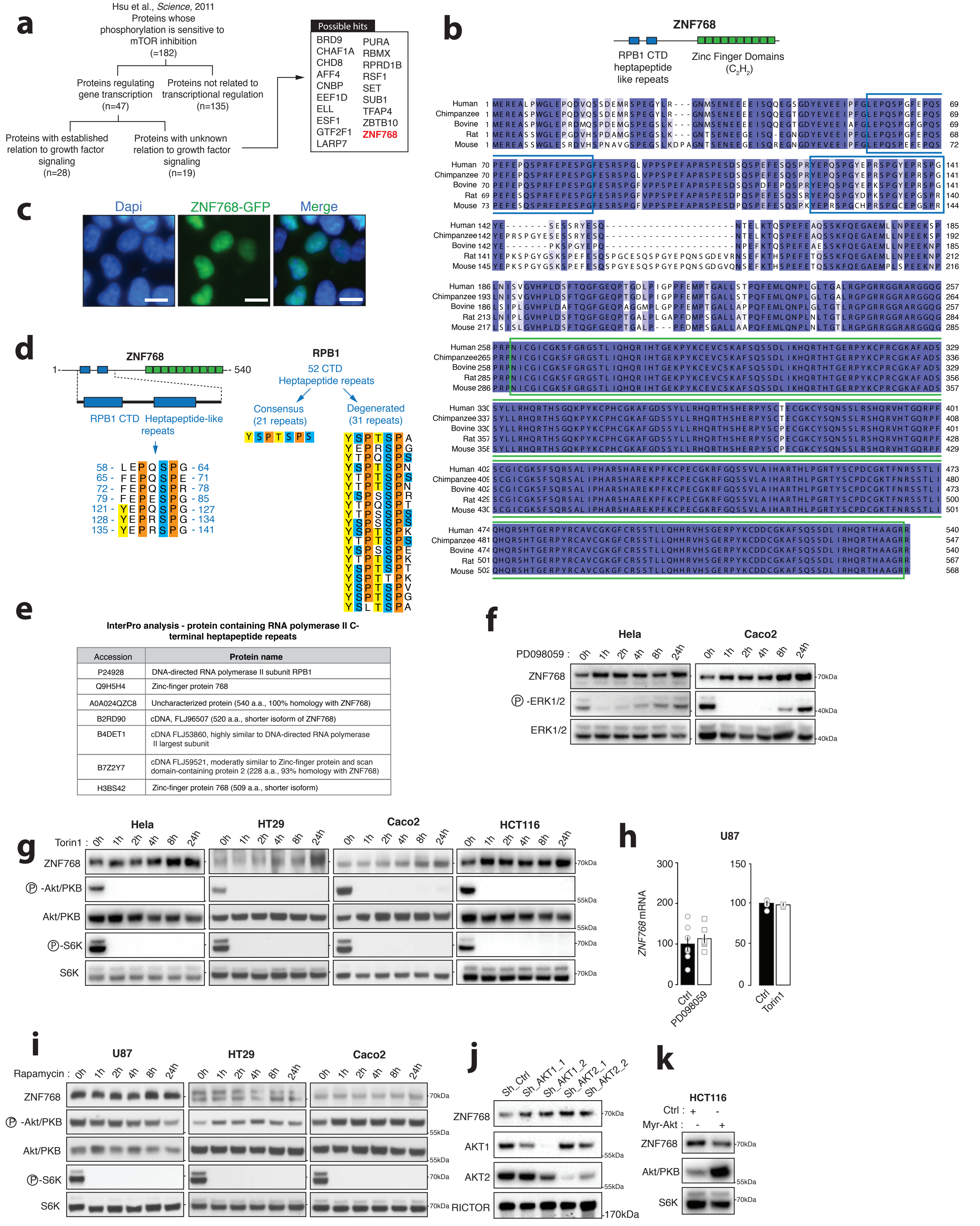

Supplementary Figure 1. Identification of ZNF768 as a phosphoprotein destabilized upon RAS activation. (A) Overview of the experimental scheme used to identify new transcriptional regulators affected by growth factor signaling. All the proteins potentially phosphorylated downstream of mTOR were extracted from the study of Hsu et al[19]. Proteins with DNA binding abilities or potential roles in transcription were selected. Of this group, proteins with unknown relation to growth factor signaling were selected as potential hits. All the proteins identified are listed in Supplementary Data 1. **(B)** ZNF768 protein sequences from several organisms were aligned. The blue squares on the N-terminal end represent the CTD domains. The green squares in the C-Terminal part mark the C2H2 domains. **(C)** GFP-ZNF768 was overexpressed in Hela cells and live imaging was performed. DAPI was used to stain the nuclei. Representative pictures are shown (Scale: 12,5µm). **(D)** Schematic presentation of ZNF768 protein. The RPB1 CTD heptapeptide-like repeats found in ZNF768 are presented. The consensus heptapeptide repeats and the 31 degenerated repeats present in RPB1 are shown. **(E)** Large-scale protein sequence analysis using InterPro indicates that ZNF768 is the only human protein beyond RPB1 that contains heptapeptide repeats. **(F-G)** Various cell lines were treated with either **(F)** PD098059 (50 µM) or **(G)** Torin1 (250 nM) for the indicated time. Protein lysates were prepared and western blots performed. **(H)** U87 cells were treated with either PD098059 (50 µM) or Torin1 (250µM) for 24h hours. *ZNF768* expression was measured by RT-qPCR (n=4 condition for Torin1 and n=7/condition for PD098059). **(I)** Cell lines were treated with Rapamycin (100µm) for the indicated time and western blots were performed. **(J)** U87 cells were transduced with lentiviruses expressing shRNA to knockdown either Akt1 or Akt2. Protein were extracted and western blots were performed. **(K)** HCT116 cells were transduced to overexpress a control protein or myr-Akt and western blots were performed. In all panels, data represent the mean ± SEM. In panel H, significance was determined by two-tailed, unpaired *t* test. No significant results were observed. Details about reproducibility are provided in the Statistics and reproducibility included in the Methods section**.** Source data are provided as a Source data file.

Supplementary Figure 2. ZNF768 depletion blocks proliferation and causes cellular senescence.

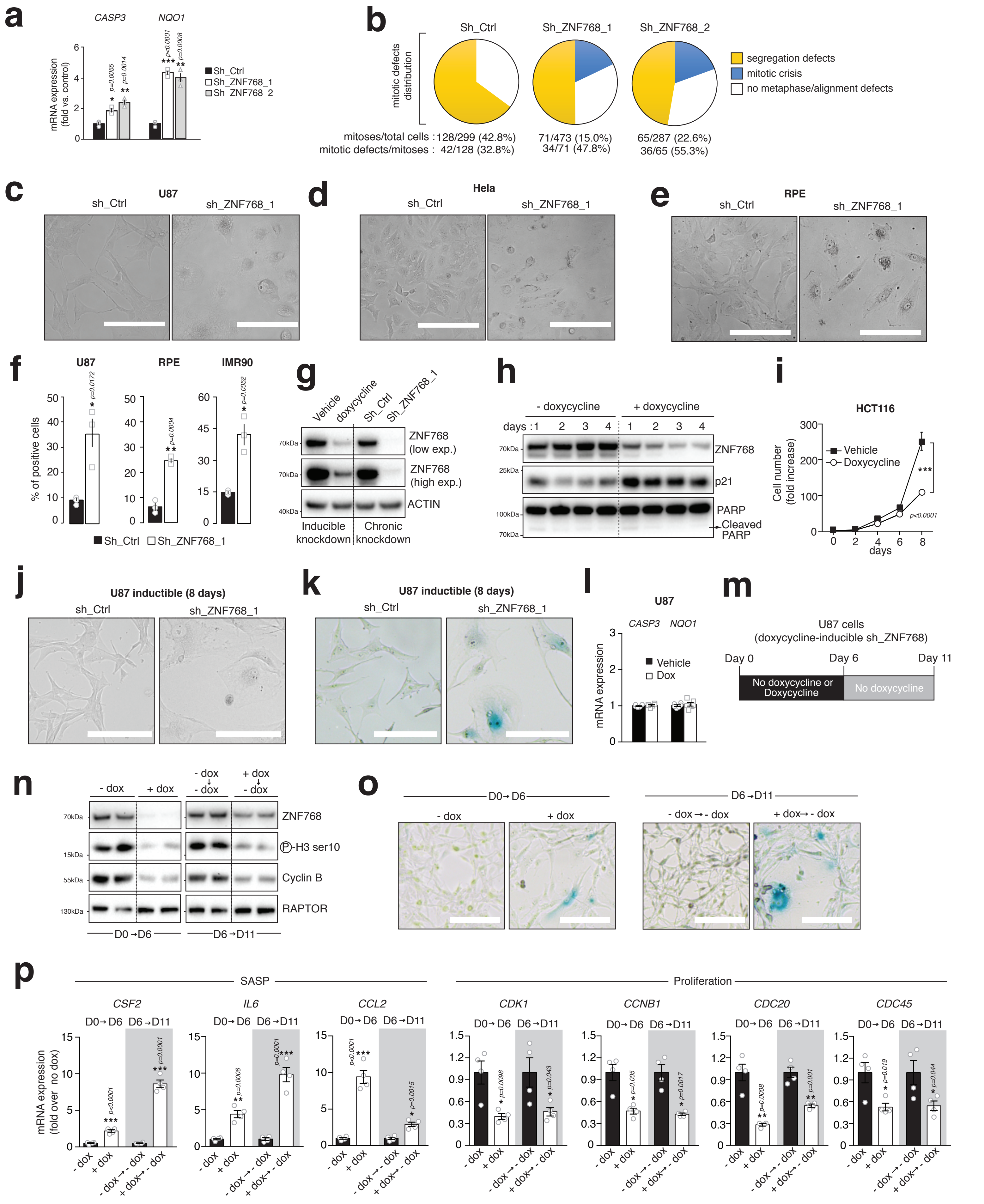

Supplementary Figure 2. ZNF768 depletion blocks proliferation and causes cellular senescence (A) U87 cells were transduced to knockdown ZNF768. Gene expression was measured by RT-qPCR (n=3/condition) 96 hours post-infection. **(B)** ZNF768 was depleted from Hela cells using shRNA. 48h post transduction, cells were stained with SiR-DNA and used in live-imaging microscopy. **(C to E)** Cells were transduced to knockdown ZNF768. Representative pictures are presented (Scale: 200µm). **(F)** SA-β-gal staining was performed following ZNF768 knockdown in U87, RPE and IMR90 cells. The % of SA-β-gal positive cells is presented (n=3/cell line). **(G)** U87 cells were transduced with lentiviruses allowing the conditional expression of a shRNA targeting ZNF768. Cells were treated with doxycycline (20 ng/µl) for 96 hours. In parallel, other U87 cells were transduced for 24 hours with lentiviruses expressing a shRNA targeting ZNF768. Cells were selected over the following 72 hours (chronic knockdown). Western blots were performed. **(H)** Inducible HCT116 cells were treated with doxycycline (20 ng/µl) to repress ZNF768 and western blots were performed. **(I)** Inducible HCT116 cells were treated or not with doxycycline (20 ng/µl) and counted (n=4). **(J)** Inducible U87 cells were treated or not with doxycycline (20 ng/µl) for 7 days to repress ZNF768 and pictures were taken. Representative images are shown (Scale: 200µm) **(K)** SA-β-gal staining was performed in the experiment described in J. Representative images are presented (Scale: 200µm). **(L)** U87 cells were treated as described in J. Gene expression was measured by RT-qPCR (n=6/condition). **(M)** Overview of the experiments presented in Supplementary Figures 2N to 2P. Inducible U87 cells were treated or not with doxycycline (20 ng/µl) for 6 days to repress ZNF768. Cells were next washed and incubated without doxycycline from day 6 to day 11. **(N)** Western blots and **(O)** SA-β-gal staining were performed from the cells described in M. Representative images are presented (Scale: 200µm). **(P)** Genes expression was measured by RT-qPCR from the experiment described in M (n=4/condition). Results are presented as fold change vs control. In all panels, data represent the mean ± SEM. In panel A, F and L and P, significance was determined by two-tailed, unpaired *t* test. In panel I, significance was determined

by Two-way ANOVA. Details about reproducibility are provided in the Statistics and reproducibility included in the Methods section. Source data are provided as a Source data file.

Supplementary Figure 3. ZNF768 depletion affects the expression of key genes controlling proliferation and senescence.

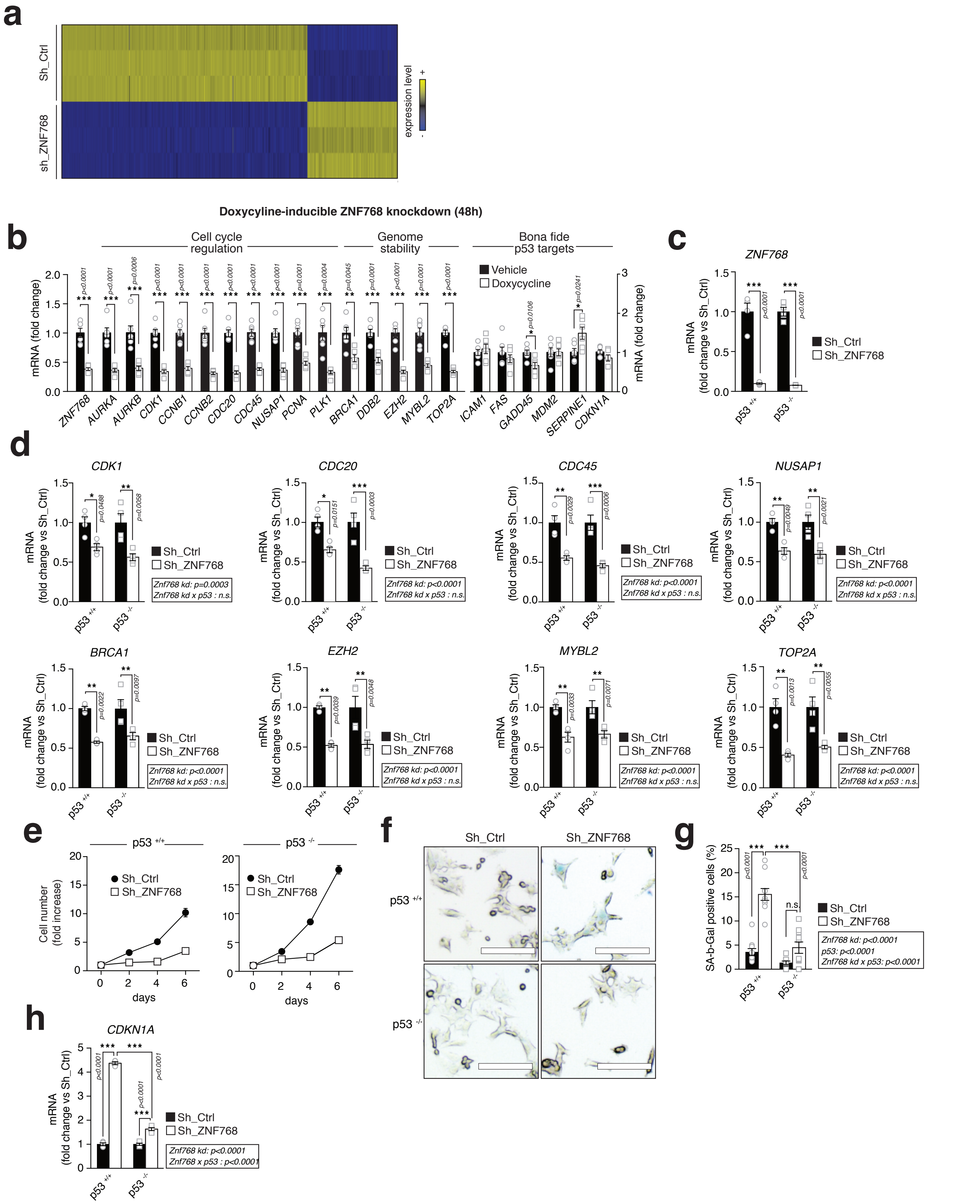

Supplementary Figure 3. ZNF768 depletion affects the expression of key genes controlling proliferation and senescence. (A) Heatmap presenting an overview of the transcriptional impact linked to ZNF768 depletion. U87 cells were transduced with lentiviruses to knockdown ZNF768 and selected. 96 hours post infection, RNA was isolated and microarrays were performed. **(B)** U87 cells were transduced with lentiviruses allowing the conditional expression of a shRNA targeting ZNF768. After selection with puromycin, cells were treated with doxycycline (20 ng/µl) for 48 hours to knockdown ZNF768. RNA was harvested and the expression of several genes was measured by RT-qPCR (n=6/condition). **(C-D)** $p53^{+/+}$ and $p53^{-/-}$ HCT116 cells were transduced with lentiviruses to deplete ZNF768. RNA was extracted RT-qPCR were performed for the indicated genes (n=4/group). For each genotype ($p53^{+/+}$ and $p53^{-/-}$), the impact of ZNF768 depletion is normalized to the control short-hairpin. **(E)** $p53^{+/+}$ and $p53^{-/-}$ HCT116 cells were transduced with lentiviruses to depleted ZNF768. 24 hours post-infection, cells were counted (day 0). Other counts were performed at day 2, 4 and 6 (n=2/condition). **(F-G)** $p53^{+/+}$ and $p53^{-/-}$ HCT116 cells were transduced with lentiviruses to depleted ZNF768. SA-β-gal staining were performed 96h post-infection. For each condition, 10 pictures were taken and analyzed from 2 independent biological measurements (n=10). Representative pictures are shown (Scale: 200µm). **(H)** RT-qPCR analyses were performed exactly as described in C (n=4/group). In all panels, data represent the mean ± SEM. In panel B, significance was determined by two-tailed, unpaired *t* test. In panels C, D, G and H, significance was determined by Two-way ANOVA with Tukey's multiple-comparisons. Details about reproducibility are provided in the Statistics and reproducibility included in the Methods section**.** Source data are provided as a Source data file.

Supplementary Figure 4. ZNF768 is depleted upon senescence entry and its overexpression contributes to bypass this process.

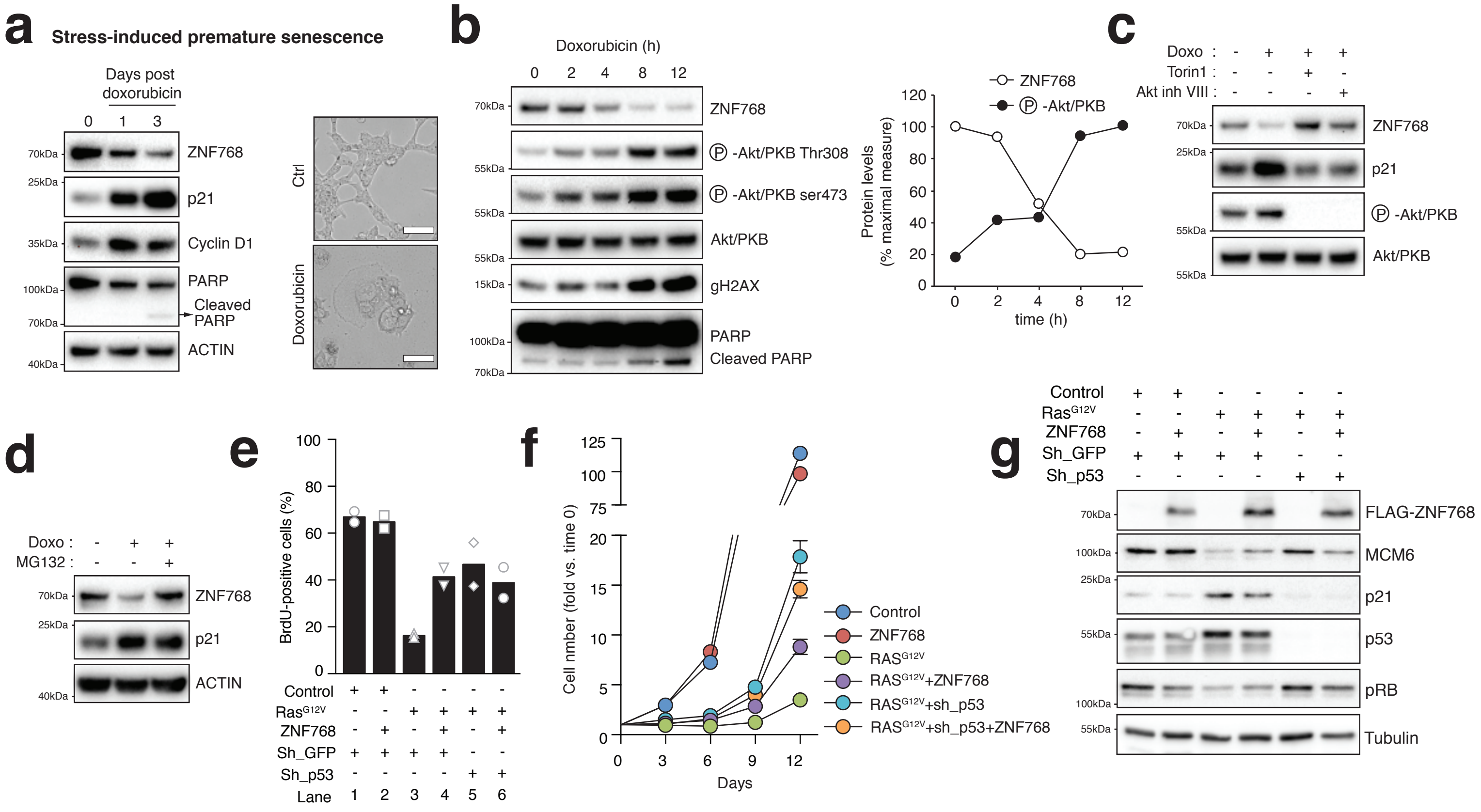

Supplementary Figure 4. ZNF768 is depleted upon senescence entry and its overexpression contributes to bypass this process. (A) HCT116 cells were treated overnight with doxorubicin (0.1 µM). The cells were next washed and followed for the indicated times. Western blot analyses were performed for the indicated proteins. Representative pictures of the cells are shown on the right part of the panel (Scale: 50µm). **(B)** HCT116 cells were serum-starved overnight and treated with doxorubicin (1 µM). Proteins were extracted and western blot analyses were performed for the indicated proteins. Quantification of ZNF768 protein and AKT phosphorylation levels calculated from the experiment is presented on the right part. **(C)** HCT116 cells were treated with doxorubicin (1 µM) in the presence of Torin1 (250 nM) or Akt inhibitor VIII (50 µM) for 6 hours. Proteins lysates were extracted for western blot analyses. **(D)** HCT116 cells expressing V5-ZNF768 were treated with 1 µM of doxorubicin for 4 hours in the presence of MG132 (20 µM). In this experiment, cells were pre-treated with MG132 2 hours prior to doxorubicin exposition. Proteins were extracted and western blots were performed for the indicated proteins. **(E)** IMR90 cells were transduced with retroviruses expressing different combinations of FLAG-ZNF768, shGFP, shp53, RAS^{G12V} or empty vector. After antibiotic selection, cells were maintained in culture for 8 days. Cells were then pulsed with BrdU for 6h prior to fixation. BrdU incorporation was detected by indirect immunofluorescence. The results presented is the average of 2 independently studies (n=2). **(F)** Cells were treated as described in E. Results represent the average of 3 independent experiments (n=3). In this panel, data represent the mean ± SEM. **(G)** Proteins were extracted from the cells described in F and western blot analyses were performed for the indicated proteins. Details about reproducibility are provided in the Statistics and reproducibility included in the Methods section**.** Source data are provided as a Source data file.

Supplementary Figure 5. ZNF768 interacts with and represses p53 transcriptional activity.

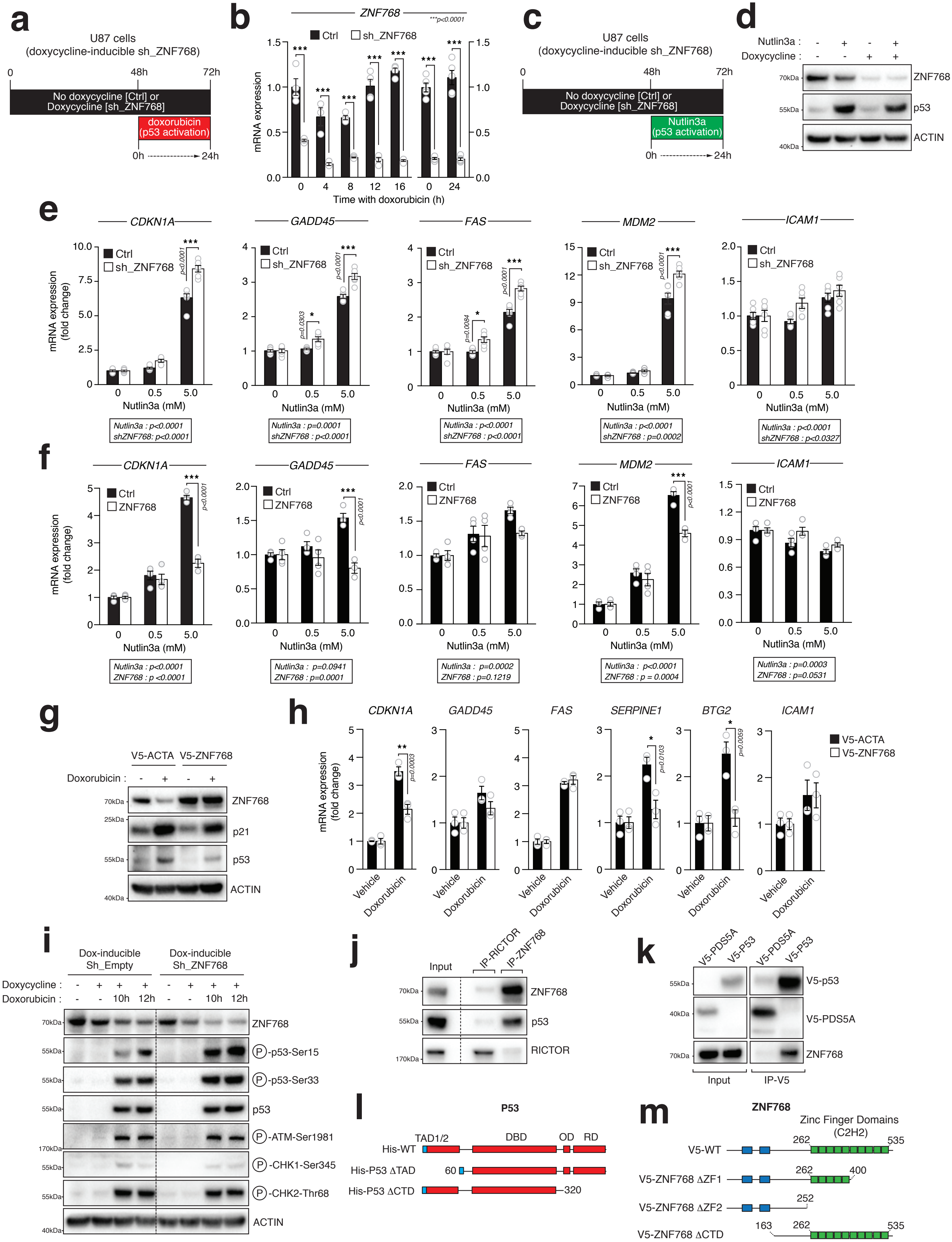

Supplementary Figure 5. ZNF768 interacts with and represses p53 transcriptional activity. (A) Overview of the experiments related to Figure 5A. U87 cells expressing a doxycycline-inducible shRNA targeting ZNF768 were treated with doxycycline (20 ng/µl, 48 hours). Cells were next treated with doxorubicin (0.5 µM) for the indicated time. **(B)** Gene expression was measured by RT-qPCR in at least 3 independent biological samples per condition (n=3/condition) from the experiment described in A. **(C)** Overview of the experiments related to Supplementary Figure 5D and 5E. U87 cells expressing a doxycycline-inducible shRNA targeting ZNF768 were treated with doxycycline (20 ng/µl, 48 hours). Cells were next treated with Nutlin3a for 24 hours. **(D)** Western blots were performed from the experiment described in C. **(E)** Gene expression was measured by RT-qPCR in at least 5 independent biological samples per condition (n=5/condition) from the experiment described in C. Results are presented as fold change over controls. **(F)** U87 cells were transduced to overexpress ZNF768 and were then treated with Nutlin3a for 24 hours. Gene expression was measured by RT-qPCR in at least 3 independent biological samples per condition (n=3/condition). Results are presented as fold change over controls. **(G-H)** HCT116 were transduced to overexpress ZNF768. Cells were treated with 0.1 µM of doxorubicin for 4 hours. The cells were next washed and protein and mRNA were extracted 48 hours later. Results are presented as fold change over controls. **(G)** Western blot analyses and **(H)** RT-qPCR analyses were next performed (n=3/condition). **(I)** HCT116 cells expressing a doxycycline-inducible shRNA targeting ZNF768 were treated with doxycycline (20 ng/µl) overnight to repress ZNF768. Cells were treated with doxorubicin (0.5 µM) and western blots were performed. **(J)** Endogenous ZNF768 or RICTOR were immunoprecipitated from 293T cells and western blots were performed. **(K)** 293T cells were transfected and V5 proteins were immunoprecipitated. Western blots were next performed. **(L-M)** Overview of the constructs used in the experiments described in **(L)** Figure 5G and **(M)** Figure 5H and I. In all panels, data represent the mean ± SEM. In panel B, E, F and H significance was determined by Two-way ANOVA with Tukey's multiple-

comparisons test. Details about reproducibility are provided in the Statistics and reproducibility included in the Methods section. Source data are provided as a Source data file.

Supplementary Figure 6. ZNF768 expression and protein levels are elevated in several human cancers.

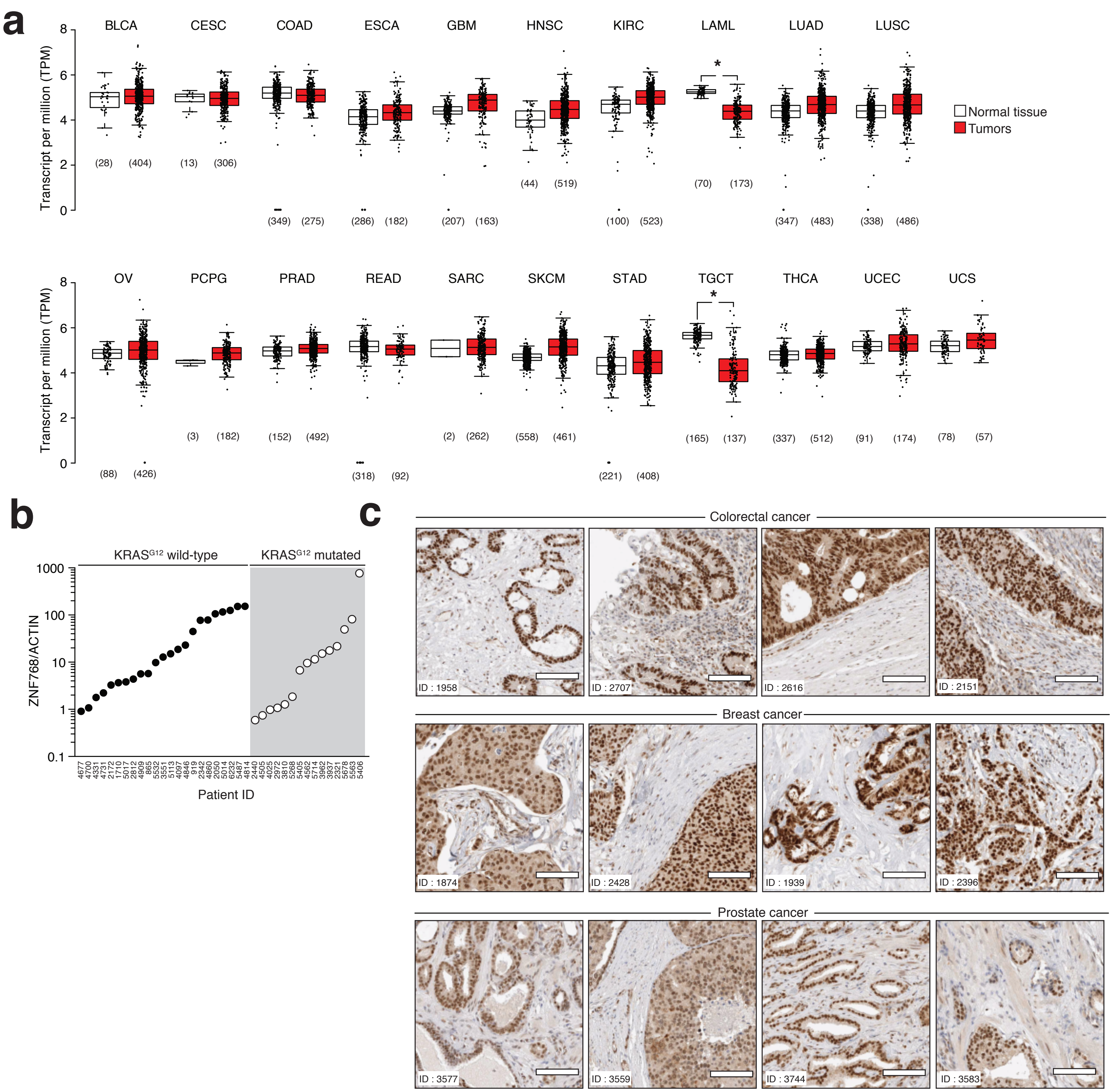

Supplementary Figure 6. ZNF768 expression and protein levels are elevated in several human cancers. (A) Gene expression analysis of ZNF768 expression between normal and cancer tissues. This analysis was performed using the GEPIA resource. The method for differential analysis is one-way ANOVA, without adjustment for multiple comparisons. The Log2 fold change cut-off was set up at 0.5 and the *p*-value cut-off at 0.001 ($^{*}p < 0.001$ versus control). Each dot represents one sample. The number (n) of sample analyzed is provided below each graph bar. The low and high limits of the box represent the lower and upper quartiles, respectively. The middle line in the box represents the median value. The whiskers are 1,5 times the inter-quartile range. **(B)** Lung tumors samples were genotyped for the presence of altered $KRAS^{G12}$ allele and sorted according to ZNF768 protein levels. ZNF768 protein levels were measured by western blot, as described in Figure 6D. **(C)** Immunohistochemistry analyses of ZNF768 protein in colorectal, breast and prostate cancer tumors. Examples of tumors showing high levels of ZNF768 are presented. The picture presented were extracted from the Human Protein Atlas resource (www.proteinatlas.org). To view a copy of the applicable licence, visit https://creativecommons.org/licenses/by-sa/3.0/. Representative pictures are shown (Scale: 100μm). Details about reproducibility are provided in the Statistics and reproducibility included in the Methods section**.**

Supplementary Table 1. Pathway analysis comparing Lung adenocarcinoma (LUAD) tumors with either low or high ZNF768 mRNA expression. High and low ZNF768 patients were stratified based on the median of ZNF768 expression value. The pathway enrichment analysis was performed by employing the gene set enrichment analysis (GSEA) method with pathways defined by the KEGG database from the MSIGDB. The difference between the two groups was assessed using Two-sided *t test*, which was used as the gene-level statistic. The enrichment score for each pathway was then computed using the GSEA method with statistical significance calculated using a permutation test (10,000 permutations) as implemented in the *piano* R package. Nominal p-values obtained for each pathway was corrected for multiple testing using the false discovery approach (FDR) approach, and a threshold of $P < 0.01$ was considered statistically significant.

Supplementary Table 2. Pathway analysis comparing Lung squamous carcinoma (LUSC) tumors with either low or high ZNF768 mRNA expression. High and low ZNF768 patients were stratified based on the median of ZNF768 expression value. The pathway enrichment analysis was performed by employing the gene set enrichment analysis (GSEA) method with pathways defined by the KEGG database from the MSIGDB. The difference between the two groups was assessed using Two-sided *t test*, which was used as the gene-level statistic. The enrichment score for each pathway was then computed using the GSEA method with statistical significance calculated using a permutation test (10,000 permutations) as implemented in the *piano* R package. Nominal p-values obtained for each pathway was corrected for multiple testing using the false discovery approach (FDR) approach, and a threshold of $P < 0.01$ was considered statistically significant.

Supplementary Table 3. List of genes commonly repressed in response to ZNF768 inhibition. This list was generated by comparing data collected from U2OS cells overexpressing a dominant negative ZNF768 (Rohrmoser et al., Nucleic acids research, (2019)) with data collected following the knockdown of ZNF768 in U87 cells (full data set presented in Supplementary Data 3).
