# Supplementary material for: ZNF768 links oncogenic RAS to cellular senescence
Source: Nat Commun. 2021 Aug 17;12:4841. doi: 10.1038/s41467-021-24932-w (PMC8370976; doi:10.1038/s41467-021-24932-w)
Supplement: Supplementary file 2 — Description of Additional Supplementary Files [file 41467_2021_24932_MOESM2_ESM.pdf]

Description of Additional Supplementary Files

File Name: Supplementary Data 1
Description: List of uncharacterized transcriptional regulators potentially phosphorylated in response to growth factor signaling.

File Name: Supplementary Data 2
Description: GO and Metascape analysis of the genes differentially expressed in 6 different cell lines using the iLincs database.

File Name: Supplementary Data 3
Description: Gene expression profile of U87 cells depleted from ZNF768.

File Name: Supplementary Data 4
Description: GO and Metascape analysis of the genes downregulated in U87 cells upon ZNF768 knockdown.

File Name: Supplementary Data 5
Description: GO and Metascape analysis of the genes upregulated in U87 cells upon ZNF768 knockdown.
