# Supplementary material for: ZNF768 links oncogenic RAS to cellular senescence
Source: Nat Commun. 2021 Aug 17;12:4841. doi: 10.1038/s41467-021-24932-w (PMC8370976; doi:10.1038/s41467-021-24932-w)
Supplement: Supplementary file 8 — Reporting Summary [file 41467_2021_24932_MOESM8_ESM.pdf]

nature research

Corresponding author(s): Mathieu Laplante

Last updated by author(s): 5/18/2021

Reporting Summary

Nature Research wishes to improve the reproducibility of the work that we publish. This form provides structure for consistency and transparency in reporting. For further information on Nature Research policies, see our Editorial Policies and the Editorial Policy Checklist.

Statistics

For all statistical analyses, confirm that the following items are present in the figure legend, table legend, main text, or Methods section.

n/a	Confirmed	
☐	☒	The exact sample size (n) for each experimental group/condition, given as a discrete number and unit of measurement
☐	☒	A statement on whether measurements were taken from distinct samples or whether the same sample was measured repeatedly
☐	☒	The statistical test(s) used AND whether they are one- or two-sided<br>*Only common tests should be described solely by name; describe more complex techniques in the Methods section.*
☐	☒	A description of all covariates tested
☐	☒	A description of any assumptions or corrections, such as tests of normality and adjustment for multiple comparisons
☐	☒	A full description of the statistical parameters including central tendency (e.g. means) or other basic estimates (e.g. regression coefficient) AND variation (e.g. standard deviation) or associated estimates of uncertainty (e.g. confidence intervals)
☐	☒	For null hypothesis testing, the test statistic (e.g. F, t, r) with confidence intervals, effect sizes, degrees of freedom and P value noted<br>*Give P values as exact values whenever suitable.*
☒	☐	For Bayesian analysis, information on the choice of priors and Markov chain Monte Carlo settings
☒	☐	For hierarchical and complex designs, identification of the appropriate level for tests and full reporting of outcomes
☒	☐	Estimates of effect sizes (e.g. Cohen's d, Pearson's r), indicating how they were calculated

Our web collection on statistics for biologists contains articles on many of the points above.

Software and code

Policy information about availability of computer code

Data collection	Mass spectra were acquired using a data dependent acquisition mode using Analyst software version 1.7.; MGF peak list files were created using Protein Pilot version 4.5 software (Sciex). MGF sample files were then analyzed using Mascot (Matrix Science, London, UK; version 2.5.1). Scaffold (version Scaffold_4.8.4, Proteome Software Inc., Portland, OR) was used to validate MS/MS based peptide and protein identification. Image acquisition was performed using Metamorph software (Molecular Devices, version 7.8.4.0). Image Lab software (version 5.1) was used to acquire and analyze western blot images.
Data analysis	Data was analyses using Prism 6.0.

For manuscripts utilizing custom algorithms or software that are central to the research but not yet described in published literature, software must be made available to editors and reviewers. We strongly encourage code deposition in a community repository (e.g. GitHub). See the Nature Research guidelines for submitting code & software for further information.

Data

Policy information about availability of data

All manuscripts must include a data availability statement. This statement should provide the following information, where applicable:

- Accession codes, unique identifiers, or web links for publicly available datasets
- A list of figures that have associated raw data
- A description of any restrictions on data availability

The source data underlying the all Figures and Supplemental Figures are included as Source Data Files. All data are available. The Uniprot HomoSapiens proteome used in mass spectrometry analyses can be found at this link https://www.uniprot.org/

Field-specific reporting

Please select the one below that is the best fit for your research. If you are not sure, read the appropriate sections before making your selection.

☒ Life sciences ☐ Behavioural & social sciences ☐ Ecological, evolutionary & environmental sciences

For a reference copy of the document with all sections, see nature.com/documents/nr-reporting-summary-flat.pdf

Life sciences study design

All studies must disclose on these points even when the disclosure is negative.

Sample size	No sample size calculations were done as the magnitude of the effect sizes were unknown. The vast majority of the experiments outlined in this manuscript utilize cell-based systems, where conditions are standard and extreme variability is decreased as a result. Thus, for most of the experiments described, we used between 3-6 independent samples for each nominal variable. This number of independent experiments is a standard sample size to accurately detect differences in cell biology and molecular biology experiments. As reference, the sample sizes were guided on the basis of similar published studies (PMID: 32612235; PMID: 32814897). Western blots are shown as representative images. Key findings were reproduced in at least 2-3 independent experiments or in different cell lines.
Data exclusions	In Figure S5E, F and H, one measurement is missing from the RT-qPCR for the analyses of the gene GADD45, MDM2 and BTG2 respectively, due to technical issues while running the PCR.
Replication	Key experiments were repeated multiple times with similar results as indicated in the manuscript. Moreover, many experiments were replicated independently in multiple cell lines to insure reproducibility and validity of the experiments. A complete description of the number of times each experiment was performed is provided in the section 'Statistics and Reproducibility' of our manuscript.
Randomization	No randomization was done for cell culture experiments. Same plate of cells were used to set up the treatment groups in each experiment.
Blinding	Blinding was not performed for the in vitro experiments. The investigators needed to know the treatment groups in order to perform the study, and the data analyses were based on objectively measurable data. The investigators were not blinded when collecting lung samples and tumors from patients. Proteins were extracted blindly from samples of normal lung and lung tumors with denominated samples. Blinding was not performed for the western blot analyses as matched samples (normal lung and tumors from each patient) had to be analyzed side by side on gels.

Reporting for specific materials, systems and methods

We require information from authors about some types of materials, experimental systems and methods used in many studies. Here, indicate whether each material, system or method listed is relevant to your study. If you are not sure if a list item applies to your research, read the appropriate section before selecting a response.

Materials & experimental systems

n/a	Involved in the study	
☐	☒	Antibodies
☐	☒	Eukaryotic cell lines
☒	☐	Palaeontology and archaeology
☒	☐	Animals and other organisms
☐	☒	Human research participants
☒	☐	Clinical data
☒	☐	Dual use research of concern

Methods

n/a	Involved in the study	
☒	☐	ChIP-seq
☒	☐	Flow cytometry
☒	☐	MRI-based neuroimaging

Antibodies

Antibodies used	The following antibodies were used: ZNF768 [Aviva Systems Biology, FLJ23436, dilution 1 :1000] ; Akt (pan) [C67E7, Cell Signaling Technology, #4691, dilution 1:1000] ; Phopho-Akt (ser473) [D9E, Cell Signaling Technology, #9271, dilution 1:1000], Phopho-Akt (Thr308) [244F9, Cell Signaling Technology, #9275, dilution 1:1000], p44/42 MAPK (Erk1/2)[137F5, Cell Signaling Technology, #9102, dilution 1:1000], Phospho-p44/42 MAPK (Erk1/2)(Thr202/Tyr204)[D13.14.4E, Cell Signaling Technology, #9101, dilution 1:1000], p70 S6 kinase [Cell Signaling Technology, #9202, dilution 1:1000], Phospho-p70 S6 Kinase (Thr389) [Cell Signaling Technology, #9205, dilution 1:1000), Raptor [24C12, Cell Signaling Technology, #2280, dilution 1:1000], Rictor [53A2, Cell Signaling Technology, #2114, dilution 1:1000), V5-Tag [D3H8Q, Cell Signaling Technology, #13202, dilution 1:1000], Caspase-3 [Cell Signaling Technology, #9662, dilution 1:1000], PARP [46D11, Cell Signaling Technology, #9532, dilution 1:1000], p21 Waf1/Cip1 [12D1, Cell Signaling Technology, #2947, dilution 1:1000], p16 INK4A [D7C1M, Cell Signaling Technology, #80772, dilution 1:1000], p53 [1C12, Cell Signaling Technology, #2524, dilution 1:1000], Phospho-p53 (Ser15) [Cell Signaling Technology, #2525, dilution 1:1000], Phospho-p53 (Ser33) [Cell Signaling Technology, #2526, dilution 1:1000], Acetyl-p53 (Lys382) [16G8, Cell Signaling Technology, #9286, dilution 1:1000], Cyclin D1 [92G2, Cell Signaling Technology, #2978, dilution 1:1000], Phospho-histone H3 (Ser10)[D2C8, Cell Signaling Technology, #3377, dilution 1:1000], Phospho-histone H2A.X (Ser139) [20E3, Cell Signaling Technology, #9718, dilution 1:1000], S6 Ribosomal

Protein [5G10, Cell Signaling Technology, #2217, dilution 1:2500], Cyclin B1 [D5C10, Cell Signaling Technology, #12231, dilution 1:1000], anti–β-actin [Cell Signaling Technology, #4967, dilution 1:1000], Phospho-ATM (ser1981) [Cell Signaling Technology, #5883, dilution 1:1000], Phospho-CHK1 (Ser345) [Cell Signaling Technology, #2348, dilution 1:1000], Phospho-CHK2 (Thr68) [Cell Signaling Technology, #2197, dilution 1:1000] FLAG [M2, Sigma Aldrich, F3165, dilution 1:1000], MCM6 [Bethyl Laboratories, # A300-194A, 1:2000], phospho-Rb (T826) [EPR5351, Abcam, # ab133446, dilution 1:1000], α-Tubulin [B-5-1-2, Sigma Aldrich, #T5168, dilution 1:20000], p21 [C-19, Santa Cruz Biotechnologies, # SC397, dilution 1:500], p53 HRP-Conjugated [R&D Systems, HAF1355, dilution 1:5000], Phospho-MAPK/CDK substrates (PXS*P or S*PXR/K) [Cell Signaling Technology, #2325, dilution 1:1000]). Alexa 488-conjugated anti-BrdU antibody (ThermoFisher Scientific B35130, 1:200),
Secondary antibodies were purchased for Cell Signaling Technology [Cell Signaling Technology, #7074, #7075, dilution 1:5000].

Validation

ZNF768 [Aviva Systems Biology, FLJ23436]. Used for WB, IP, IHC. Species : Human. The validity of the antibody was tested in response to i) ZNF768 knockdown and ii) ZNF768 overexpression in mouse and human cells. Tissues from ZNF768 KO mice were used to validate the IHC.
Akt (pan) [C67E7, Cell Signaling Technology, #4691.] Used for WB. Species : Human. The antibody was validated by the company and by users (2681 citations)
Phospho-Akt (ser473) [D9E, Cell Signaling Technology, #9271]. Used for WB. Species : Human. The antibody was validated by the company and by users (5197citations)
Phospho-Akt (Thr308) [244F9, Cell Signaling Technology, #9275]. Used for WB. Species : Human. The antibody was validated by the company and by users (1024 citations)
p44/42 MAPK (Erk1/2)[137F5, Cell Signaling Technology, #9102]. Used for WB. Species : Human. The antibody was validated by the company and by users (4986 citations)
Phospho-p44/42 MAPK (Erk1/2)(Thr202/Tyr204)[D13.14.4E, Cell Signaling Technology, #9101]. Used for WB. Species : Human. The antibody was validated by the company and by users (5554 citations)
p70 S6 kinase [Cell Signaling Technology, #9202]. Used for WB. Species : Human. The antibody was validated by the company and by users (1245 citations)
Phospho-p70 S6 Kinase (Thr389) [Cell Signaling Technology, #9205]. Used for WB. Species : Human. The antibody was validated by the company and by users (1177 citations)
Raptor [24C12, Cell Signaling Technology, #2280]. Used for WB. Species : Human. The antibody was validated by the company and by users (360 citations)
Rictor [53A2, Cell Signaling Technology, #2114]. Used for WB, IP Species : Human. The antibody was validated by the company and by users (145 citations)
V5-Tag [D3H8Q, Cell Signaling Technology, #13202]. Used for WB, IP Species : Human. The antibody was validated by the company and by users (126 citations)
Caspase-3 [Cell Signaling Technology, #9662]. Used for WB. Species : Human. The antibody was validated by the company and by users (2723 citations)
PARP [46D11, Cell Signaling Technology, #9532]. Used for WB. Species : Human. The antibody was validated by the company and by users (970 citations)
p21 Waf1/Cip1 [12D1, Cell Signaling Technology, #2947]. Used for WB. Species : Human. The antibody was validated by the company and by users (1126 citations)
p16 INK4A [D7C1M, Cell Signaling Technology, #80772]. Used for WB. Species : Human. The antibody was validated by the company and by users (29 citations)
p53 [1C12, Cell Signaling Technology, #2524]. Used for WB, IP Species : Human. The antibody was validated by the company and by users (873 citations)
Phospho-p53 (Ser15) [Cell Signaling Technology, #2525] Used for WB Species : Human. The antibody was validated by the company and by users (245 citations)
Phospho-p53 (Ser33) [Cell Signaling Technology, #2526] Used for WB Species : Human. The antibody was validated by the company and by users (19 citations)
Acetyl-p53 (Lys382) [16G8, Cell Signaling Technology, #9286] Used for WB Species : Human. The antibody was validated by the company and by users (345 citations)
Cyclin D1 [92G2, Cell Signaling Technology, #2978]. Used for WB. Species : Human. The antibody was validated by the company and by users (804 citations)
Phospho-histone H3 (Ser10) [D2C8, Cell Signaling Technology, #3377]. Used for WB. Species : Human. The antibody was validated by the company and by users (245 citations)
Phospho-histone H2A.X (Ser139) [20E3, Cell Signaling Technology, #9718]. Used for WB. Species : Human. The antibody was validated by the company and by users (1031 citations)
S6 Ribosomal Protein [5G10, Cell Signaling Technology, #2217]. Used for WB. Species : Human. The antibody was validated by the company and by users (1336 citations)
Cyclin B1 [D5C10, Cell Signaling Technology, #12231]. Used for WB. Species : Human. The antibody was validated by the company and by users (129 citations)
β-actin [Cell Signaling Technology, #4967]. Used for WB. Species : Human. The antibody was validated by the company and by users (2042 citations)
Phospho-ATM (ser1981) [Cell Signaling Technology, #5883]. Used for WB. Species : Human. The antibody was validated by the company and by users (131 citations)
Phospho-CHK1 (Ser345) [Cell Signaling Technology, #2348], Used for WB. Species : Human. The antibody was validated by the company and by users (485 citations)
Phospho-CHK2 (Thr68) [Cell Signaling Technology, #2197] Used for WB. Species : Human. The antibody was validated by the company and by users (194 citations)
FLAG [M2, Sigma Aldrich, F3165]. Used for WB. Species : Human. The antibody was validated by the company and by users (5347 citations)
MCM6 [Bethyl Laboratories, # A300-194A, 1:2000]. Used for WB. Species : Human. The antibody was validated by the company and by users (9 citations)
phospho-Rb (T826) [EPR5351, Abcam, # ab133446]. Used for WB. Species : Human. The antibody was validated by the company and by users (5 citations)
α-Tubulin [B-5-1-2, Sigma Aldrich, #T5168]. Used for WB. Species : Human. The antibody was validated by the company and by users (2643 citations)
p21 [C-19, Santa Cruz Biotechnologies, # SC397]. Used for WB. Species : Human. The antibody was validated by the company and by

users (1001 citations)
p53 HRP-Conjugated [R&D Systems, HAF1355]. Used for WB. Species : Human. The antibody was validated by the company and by users (9 citations)
Phospho-MAPK/CDK substrates (PXS*P or S*PXR/K) [Cell Signaling Technology, #2325]. Used for IP. Species : Human. The antibody was validated by the company and by users (9 citations)
Alexa 488-conjugated anti-BrdU antibody [ThermoFisher Scientific B35130]. Used for IF. Species : Human. The antibody was validated by the company and by users (9 citations)

Eukaryotic cell lines

Policy information about cell lines

Cell line source(s)	All the cell lines (RPE1, Hela, U87, HCT116, MDAMB231, HEK 293, Caco-2, HT29, IMR90) were obtained from American Type Culture Collection (ATCC) or Coriell Institute and cultured according to standard mammalian tissue culture protocols and sterile technique. MEFs were produced from our internal mouse colony.
Authentication	No authentication was performed by the authors of this manuscript.
Mycoplasma contamination	All cell lines were routinely tested for mycoplasma and were at all times mycoplasma negative.
Commonly misidentified lines (See ICLAC register)	No commonly misendentified cell lines were used in our study.

Human research participants

Policy information about studies involving human research participants

Population characteristics	The patients included in this study were diagnosed with either lung adenocarcinoma (LUAD) or lung squamous cell carcinoma (LUSC) and underwent surgical resection at the Institut universitaire de cardiologie et de pneumologie de Québec – Université Laval (IUCPQ-UL). 38 patients were included in this study (19 men [14 with LUAD; 5 with LUSC] and 19 women [14 with LUAD; 5 with LUSC]. The average age of the men included in this study was 67.9+/-1.9y. The average age of the women included in this study was 64.6+/-1.6y.
Recruitment	The patients included in this study were diagnosed with either lung adenocarcinoma (LUAD) or lung squamous cell carcinoma (LUSC) and underwent surgical resection at the Institut universitaire de cardiologie et de pneumologie de Québec – Université Laval (IUCPQ-UL). All participants provided written and informed consent. There was no apparent biases in the recruitment of the patients.
Ethics oversight	The Research Ethics Committee of IUCPQ-UL approved this study (#2017-2829, 21441).

Note that full information on the approval of the study protocol must also be provided in the manuscript.
